# Use of Biocompatible Sorafenib-gold Nanoconjugates for Reversal of Drug Resistance in Human Hepatoblatoma Cells

**DOI:** 10.1038/s41598-017-08878-y

**Published:** 2017-08-17

**Authors:** Sandeep Kumar Vishwakarma, Priyanka Sharmila, Avinash Bardia, Lakkireddy Chandrakala, N. Raju, G. Sravani, B. V. S. Sastry, Md Aejaz Habeeb, Aleem Ahmed Khan, Marshal Dhayal

**Affiliations:** 1 Clinical Research Facility, Medical Biotechnology Complex, CSIR-Centre for Cellular and Molecular Biology, Uppal Road, Habsiguda, Hyderabad, 500007 Telangana India; 2 Central Laboratory for Stem Cell Research and Translational Medicine, Centre for Liver Research and Diagnostics, Deccan College of Medical Sciences, Hyderabad, 500058 Telangana India; 3 School of Biomedical Engineering, Indian Institute of Technology (Banaras Hindu University), Varanasi, 221005 India

## Abstract

The present study identifies the potential of highly biocompatible SF-GNP nano-conjugate to enhance the chemotherapeutic response to combat drug resistance in cancer cells. We developed a stable colloidal suspension of sorafenib-gold nanoconjugate (SF-GNP) of <10 nm size in aqueous medium for reverting the cancer drug resistance in SF-resistant HepG2 cells in a 3D *ex-vivo* model system. *In-vivo* biocompatibility assay of SF-GNPs showed absence of systemic toxicological effects including hematological, biochemical and histological parameters. More importantly, the histopathological analysis of vital organs such as liver, brain, lung, kidney and heart showed very least or no sign of inflammation, cell infiltration, necrosis, tissue disorganization or fibrotic reactions after intra-peritoneal administration of SF-GNP nanoconjugates in animals. However, SF-GNP nanoconjugates significantly reduced (>80%) the percentage cell survival and the size and number of SF resistant solid tumor colonies of HepG2 cells in 3D model system. The exposure of SF-GNP nanoconjugate to SF resistant HepG2 cell colonies also provided evidence for anti-proliferative effect and reversal of drug resistance by elucidating the molecular regulatory mechanisms of extracellular matrix factor (CD147), tumor growth factor (TGF-β), hepatoma upregulated protein (hURP) and drug transporter (ABCG-2).

## Introduction

Drug resistance in cancer, particularly in hepatocellular carcinoma is a major delimiting factor in treatment^1, 2^. Despite the availability of a wide range of therapeutic molecules with different molecular structures and cellular targets, an overall increase in multiple drug resistance (MDR) has been observed in cancer cells^3^. Elevated expression of cell-membrane transporters, specifically ATP-binding cassette (ABC) transporters has been shown as one of the major factors responsible for drug resistance which works through the efflux of the cytotoxic dose resulting in decreased intracellular drug uptake^4^. The use of nanoparticle-based delivery systems have demonstrated the potential to overcome drug efflux mechanisms and delivery barriers in solid tumors due to enhanced permeability and retention (EPR) effect over the conventional drugs^5^. Additionally, among choices of nano-carriers^6^, the use of gold nanoparticles (GNPs) may have better promises due to its relatively higher stability and ease of functionalization. However, the biological toxicity of nanoparticles has shown a wide range of variations depending upon the synthesis condition, type of solvent used, the chemical nature of stabilizing molecules, and size variation^7–9^. Thus, the clinical applicability of reported nano-drug-delivery systems has been limited due to variability and unpredictability of their cytotoxic effects.

In present study, we aimed to develop a biologically compatible nanoconjugate of drug with GNPs which has ability to bypass efflux signaling pathways by a passive diffusion process in solid tumor model system of HepG2 cells. To insure the safely of drug-nanoconjugate, we avoided the use of organic solvents during synthesis process. Among various molecular targeted drugs (MTDs), we have chosen a multikinase inhibitor sorafenib (**SF**), the only United States Food and Drug Administration (USFDA) approved drug for treatment of hepatocellular carcinoma patients^10^ which has showed an approximately 40% of overall survival of advanced HCC patients^11^. Thus, the SF-GNPs nanoconjugates has been developed and effects of these on SF resistant HepG2 cells in solid tumor model system was studied. The major objectives for the preparation of SF-GNP nanoconjugates was to reduce systemic *in-vivo* toxicity and combat the resistance in cancer cells by regulating the expression of cancer molecules and drug efflux mechanisms.

## Results

### Synthesis of SF-GNP nanoconjugates

**Figure 1:**
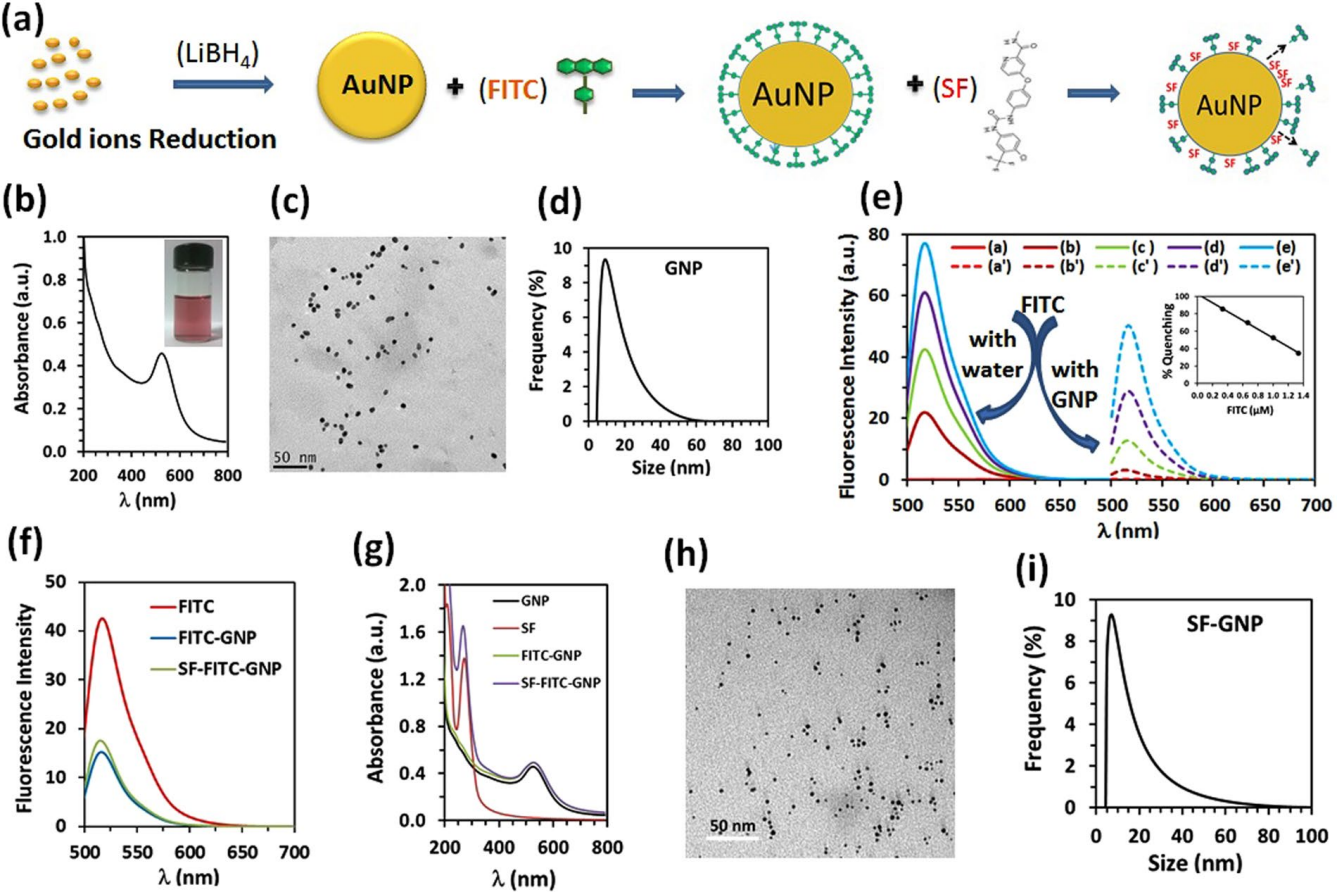
SF-GNP nano-conjugate formation and characterization of size and surface charge. (**a**) Schematic representation for synthesis of stable colloidal suspension of GNP without the use of stabilizing agent and SF-GNP nano-conjugatges, (**b**) UV-vis spectra of GNP and optical image of GNP colloidal suspension in aqueous medium, (**c**) TEM image of synthesized GNP, (**d**) DLS histogram of synthesized GNP (**e**) Quantification of FRET process between FITC and GNP at various concentration, (**f**) Fluorescence spectra of FITC, FITC-GNP and SF-FITC-GNP, (**g**) UV-vis spectra of FITC, FITC-GNP and SF-FITC-GNP, (**h**) TEM image of SF-GNP nano-conjugates, (**i**) DLS histogram of SF-GNP nano-conjugates.

Using one step process in facile hydrosol approach, synthesis of colloidal suspension of GNP was carried out in an aqueous medium^12^. The spectral confirmation of GNP was done by measuring strong Surface Plasmon Resonance (SPR) peak at 524 nm in UV-vis absorption spectra (Fig. 1b) with a very good colloidal stability due to anion capping of boron based ions^13^. The average ∼7 nm particle size of synthesized GNP in aqueous medium was obtained through TEM analysis (Fig. 1c) which was further confirmed with hydrodynamic radius measurements (Fig. 1d).

Interaction of SF with GNP was optimized by preparing fluorescein isothiocynate (FITC) functionalized GNP. When the FITC fluorescence quenched the nano-probe^14^, then SF was added which replaced the FITC into the GNP showing reappearance of fluorescence. A schematic representation for the preparation of FITC-GNP, and further to study the interaction of SF with GNP during the synthesis of SF-GNP nanoconjugates is shown in Fig. 1a. The amount of FITC fluorescence reappearance directly corresponds with the amount of SF binding on GNP surface. For this, a linearity response of various concentrations of FITC fluorescence in water was standardized (Fig. S1.2) and fluorescence resonance energy transfer (FRET) was optimized due to FITC electrostatic interactions with GNPs (Fig. S1.3). Percentage yield of FITC quenching by GNP was estimated by measuring the relative decrease in the fluorescence peak intensity of FITC in GNP colloidal suspension. The absence of FITC characteristic peaks at lower concentrations of FITC confirms the functionalization of FITC by the attachment of -NCS group of FITC on GNP surface (Fig. S1.3). A blue shift in the emission peak due to FITC (-NCS group) interaction on GNP surface and electron-hole transportation between FITC and GNP in FITC-GNP nanoconjugate showed significant reduction in fluorescence peak intensity by fluorescence quenching (Fig. 1e). UV-vis spectra of FITC showed strong absorbance at 275, 255 and 478 nm in water whereas a red shift in the absorption peak of GNP in FITC-GNP UV-vis spectra was observed with an increase in concentration of FITC.

Chemical identity of SF was established with 272 nm absorption peak in UV-vis spectra. SF interaction with FITC-GNP resulted in a blue shift of 5 nm in the SF characteristic absorption peak at 272 nm and the binding with GNP was quantified by measuring the fluorescence (Fig. 1f) and UV-vis spectra (Fig. 1g) of FITC-GNP nanoconjugate. Different concentrations (2–200 µg) of SF added in 3 ml of colloidal suspensions having 0.667 µl of FITC functionalized on GNP and corresponding increase in the fluorescence peak of FITC measured. The increase in the SF concentration in FITC-GNP colloid exhibited a relative increase in fluorescence of FITC at 520 nm which was indicative of release of FITC from FITC-GNP and formation of SF-GNP nanoconjugates. The binding kinetics of SF was optimized with addition of various concentrations of SF with FITC-GNP (Fig. S1.5). At optimum condition, stock solution of 0.33 mg/ml SF was added in GNPs colloidal suspension for preparation of SF-GNP nanoconjugates in 1:2 ratio for further experiments. The reappearance of fluorescence spectra was used as an evidence to confirm SF-GNP conjugation represented by the increase in SF concentration with gradual increase in FITC-GNP fluorescence. The average size of ∼8 nm for SF-GNP nanoconjugates was observed in TEM (Fig. 1h) which was further confirmed by the dynamic light scattering measurement (Fig. 1i).

**Figure 2:**
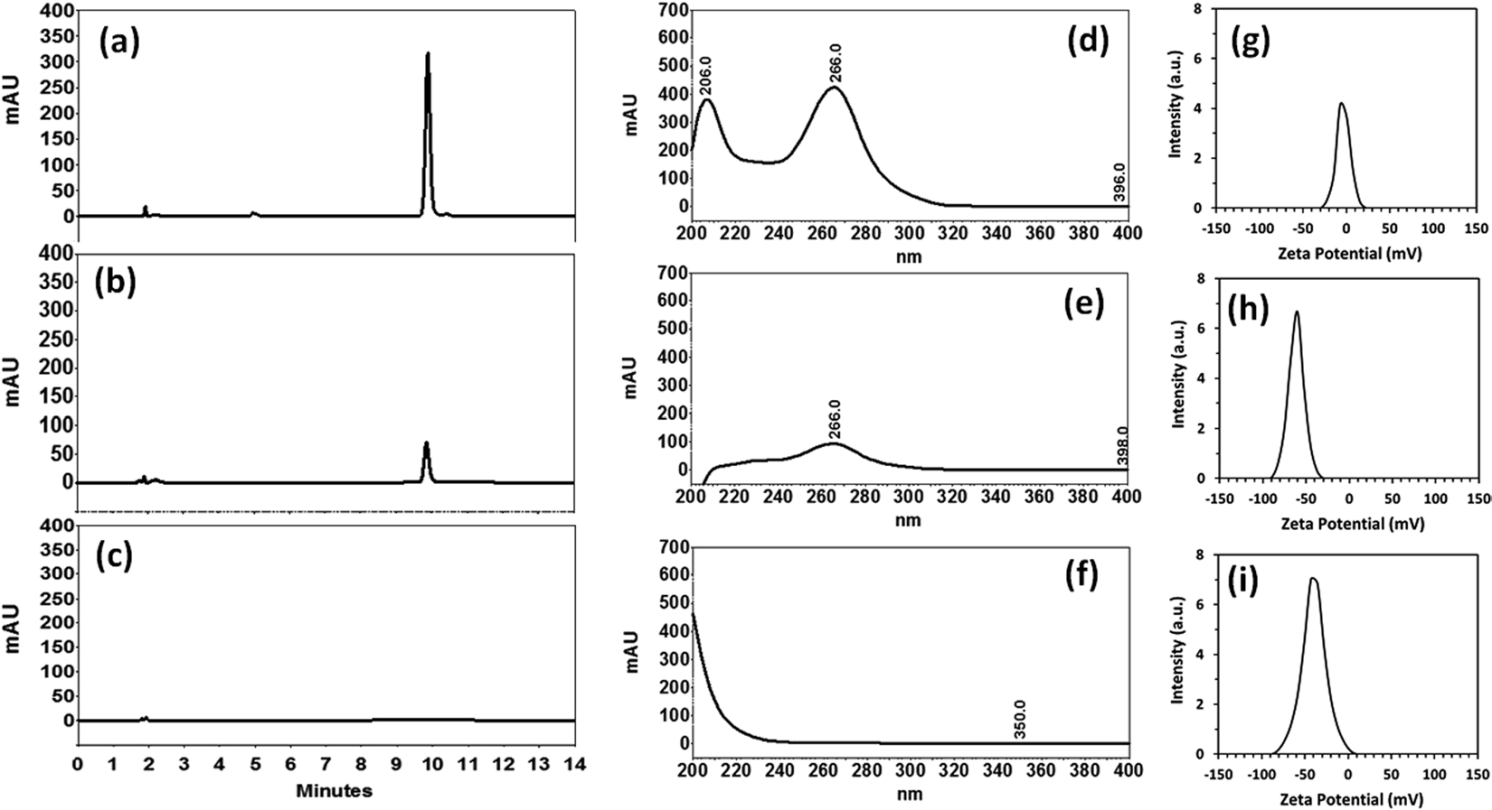
HPLC chrometophraph for (**a**) SF, (**b**) SF-GNP nano-conjugates and (**c**) GNP. UV-vis spectra of HPLC peak corresponds to (**d**) SF, (**e**) SF-GNP nano-conjugates and (**f**) GNP. Z-potential of (**g**) GNP, (**h**) SF and (**i**) SF-GNP nano-conjugates.

Further the tagging of SF on GNP was confirmed with High-performance liquid chromatography (HPLC) analysis. HPLC measurement showed the quantitative binding of SF on GNP surface. The peak area correspond to SF was calculated and a liner response with an increase in the SF concentration was observed (Table S1.1). Linearity of the HPLC method was accomplished from the correlation coefficient of calibration curve of SF which was constructed at concentration ranges of 1–1000 µg/ml. Quantitative estimation of the amount of SF binding to GNP was performed through HPLC by comparing the HPLC chromatograms of 50 μl of (~1 mg/ml) of SF solutions prepared in water and similar concentration of SF-GNP nanoconjugate. The recorded chromatogram in HPLC showed 9.86 min retention time for SF which corresponds to UV-vis spectra of SF at 255 nm (Fig. 2a) in a 14 min run time. HPLC chromatogram of SF-GNP nanoconjugate having a final concentration of ~1 mg/ml showed a small peak at 9.86 min retention time which corresponds to SF absorbance peak at 255 nm in UV-vis spectra (Fig. 2b). At similar conditions, 50 μl GNP did not show any spectral peak in the chromatogram which corresponds to SF (Fig. 2c). Percentage of SF loaded onto GNPs was measured by the relative decrease in the area of free SF with respect to the area found in the corresponding same concentration of SF loaded on GNP in aqueous colloidal suspension. SF-GNP nanoconjugates were prepared by adding SF, having a final concentration of approximately 1 mg/ml in GNPs colloidal suspension. SF-GNP peak corresponds to 255 nm absorbance in UV-vis spectra and for HPLC elutes at 9.86 min with approximately 80% reduction in the peak area as compared to the free SF of same concentration. Thus, we were able to achieve 80% of SF loading on GNP in SF-GNP nanoconjugate which has been used as optimum condition for further experiments.

The zeta potential of SF-GNP nanoconjugate was found to be ~63 mV (Fig. 2h) with decrease in negative potential in reference to GNP (−43 mV) (Fig. 2i) and only SF (~3 mV) (Fig. 2g) which has vital significance in uptake of drug within the cellular system. Negative change at the surface of nanoconjugates provides better stability in colloidal suspension.

### *In-vivo* biocompatibility of GNP and SF-GNP nanoconjugates

**Figure 3:**
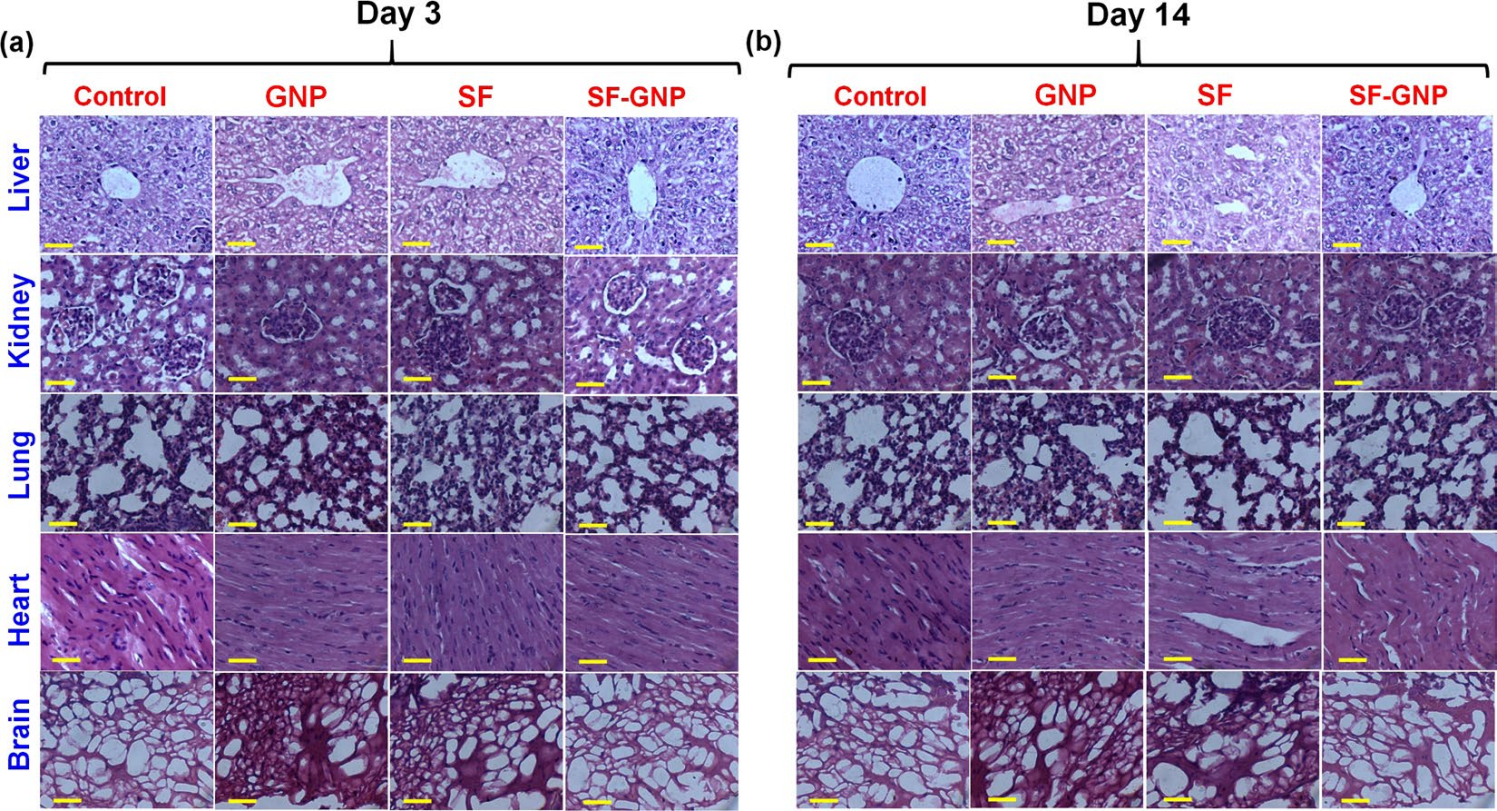
Histopathological analysis of liver, kidney, lung, heart and brain tissues was performed after H&E staining at (**a**) day 3 and (**b**) day 14. Liver and renal histology didn’t show abnormality in tissue structural organization, necrosis, fibrotic reactions or cell infiltration. The tissue histology of lung revealed no evidence of inflammation or hemorrhage, cell infiltration and changes in alveolar wall thickness. Heart tissues showed absence of major adverse effect reported in form of no sign for nuclear centralization, hemorrhage, tissue shrinkage or any adjacent fatty tissue deposition. In addition, the brain tissue from cortical region also didn’t show any significant change in organization of extracellular matrix and cellular components. (Scale bar: 20 µm, magnification: 40X).

Over the past decade, the wider acceptance of nano-delivery systems as treatment modalities has still been delimited due to the potential risk on human health despite of several advantages that have been projected in clinical translation^15^. Hence, the *in-vivo* biocompatibility of SF-GNPs nanoconjugates was assessed in male Wistar Rats (average body weight 140 ± 25 g). The serum biochemical markers specific to the liver (Albumin, Bilirubin, SGPT, SGOT and ALT) and renal function (Urea and Creatinine) were found to be in normal range demonstrating that intra-peritoneal (IP) administration of SF-GNP nanoconjugate does not alter the vital functional parameters (Fig. S2.4). Similarly the hematological parameters confirmed the normal range of blood components (WBC, RBC, Neutrophils, Basophils, Eosinophils, Monocytes, Lymphocytes, Hemoglobin, HCT and Platelets) at day 3 (Table S2.1), day 7 (Table S2.2) and day 14 (Table S2.3) post IP administration of SF-GNP nanoconjugate. These results clearly shows that neither GNP nor SF-GNP nanoconjugate produces any sort of interference in leukocytes, endothelial cells, RBCs, WBCs, neutrophils, basophils, eosinophils, monocytes, platelets count, HCT or hemoglobin quantity without hemolysis post IP exposure. This demonstrates the interaction of GNP and SF-GNP nanoconjugate with immunological and vessel wall cells does not cause cell death and interference in leukocyte-endothelial interactions and neutrophil locomotion into the tissue. Further the histopathological analysis for vital organs such as Liver, Brain, Lung, Kidney and Heart at day 3 (Fig. 3a) and day 14 (Fig. 3b) provides evidence for the absence of inflammation or hemorrhage, cell infiltration, necrosis, tissue disorganization or fibrotic reactions due to IP administration of SF-GNP nano-conjugates. More specifically, the histopathological analysis of lung revealed no evidence of inflammation or hemorrhage, cell infiltration and changes in alveolar wall thickness. Similarly, heart tissues also showed no adverse effect reported in form of absence of nuclear centralization, hemorrhage, tissue shrinkage or sign of any adjacent fatty tissue. Renal histology showed no sign of inflammation, necrosis or any other tissue abnormality. Liver histology also didn’t show abnormality in tissue structural organization, necrosis, fibrotic reactions or cell infiltration. In addition, the brain tissue from cortical region also didn’t show any significant change in organization of extracellular matrix and cellular components. This analysis revealed the biocompatibility and least systemic adverse response in vital organs against GNP and SF-GNP. The biocompatibility results presented herein for different *in-vivo* serum, blood and tissue analysis encourages for the safe applicability of GNP and SF-GNP nanoconjugates in biological system.

### SF-GNP nanoconjugates for effective inhibition of hepatobalstoma (HepG2) cells

**Figure 4:**
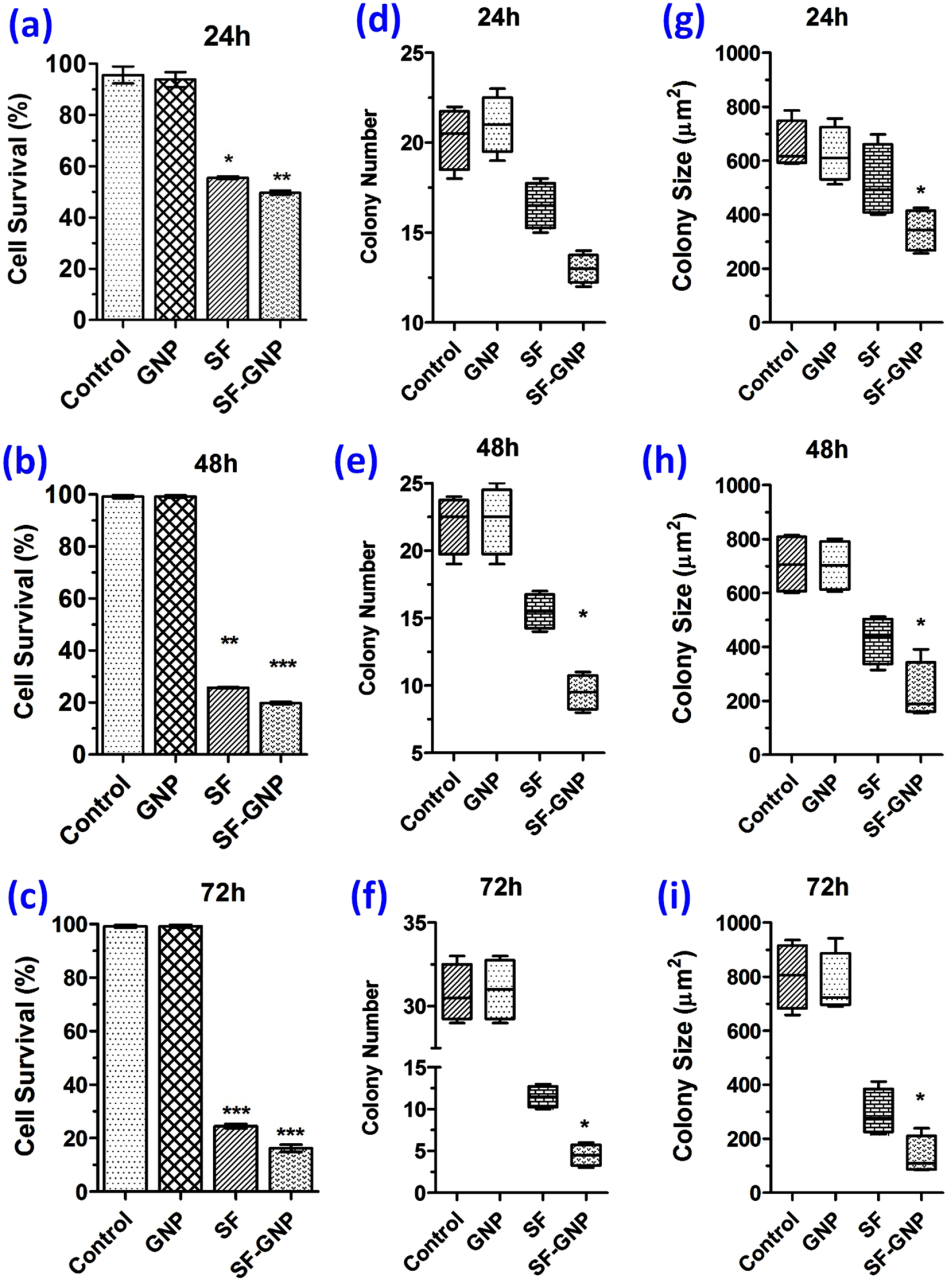
The effect of SF-GNP conjugate showed much higher response for SF-GNP as compared to the similar dose of SF and GNP on HepG2 cells analyzed at day 1, 2 and 3 in solid colonies. The mitochondrial oxidation assay revealed no different in percentage cell survival with exposure to GNPs, whereas exposure to free SF resulted in almost 30% decrease in cell survival at (**a**) day 1 (p < 0.01), (**b**) approximately 65% at day 2 (p < 0.001) and (**c**) almost 70% at day 3 (p < 0.001). Interestingly, the treatment with similar dose of SF-GNP conjugate enhanced chemotherapeutic efficacy resulting in approximately 5–10% higher cell death as compared to free SF at respective time points (day 1: p < 0.01, day 2 (p < 0.01). Further the effect of SF-GNP conjugate was identified by analyzing the size and number of solid HepG2 cell colonies in semi solid agar medium which showed gradual decrease in colony number and colony size with SF-GNP treatment as compared to free SF at (**d**,**g**) day 1 (**e**,**h**) day 2 (p < 0.01) and (**f**,**i**) day 3 (p < 0.01).

The impelled therapeutic efficacy of SF-GNP nanoconjugate was evaluated on hepatoblastoma (HepG2) cells and correlated with the effect of free SF on inhibition of HepG2 cell proliferation at day 1 (Fig. 4a), day 2 (Fig. 4b) and day 3 (Fig. 4c). There was no significant inhibition in the cancer cell growth with the use of GNPs whereas free SF showed ∼70% inhibition after Day 3 post treatment. Interestingly, SF-GNP nanoconjugates further impelled the enhanced chemotherapeutic efficacy > 80% and less than 10% of the cancer cell survived after Day 3.

**Figure 5:**
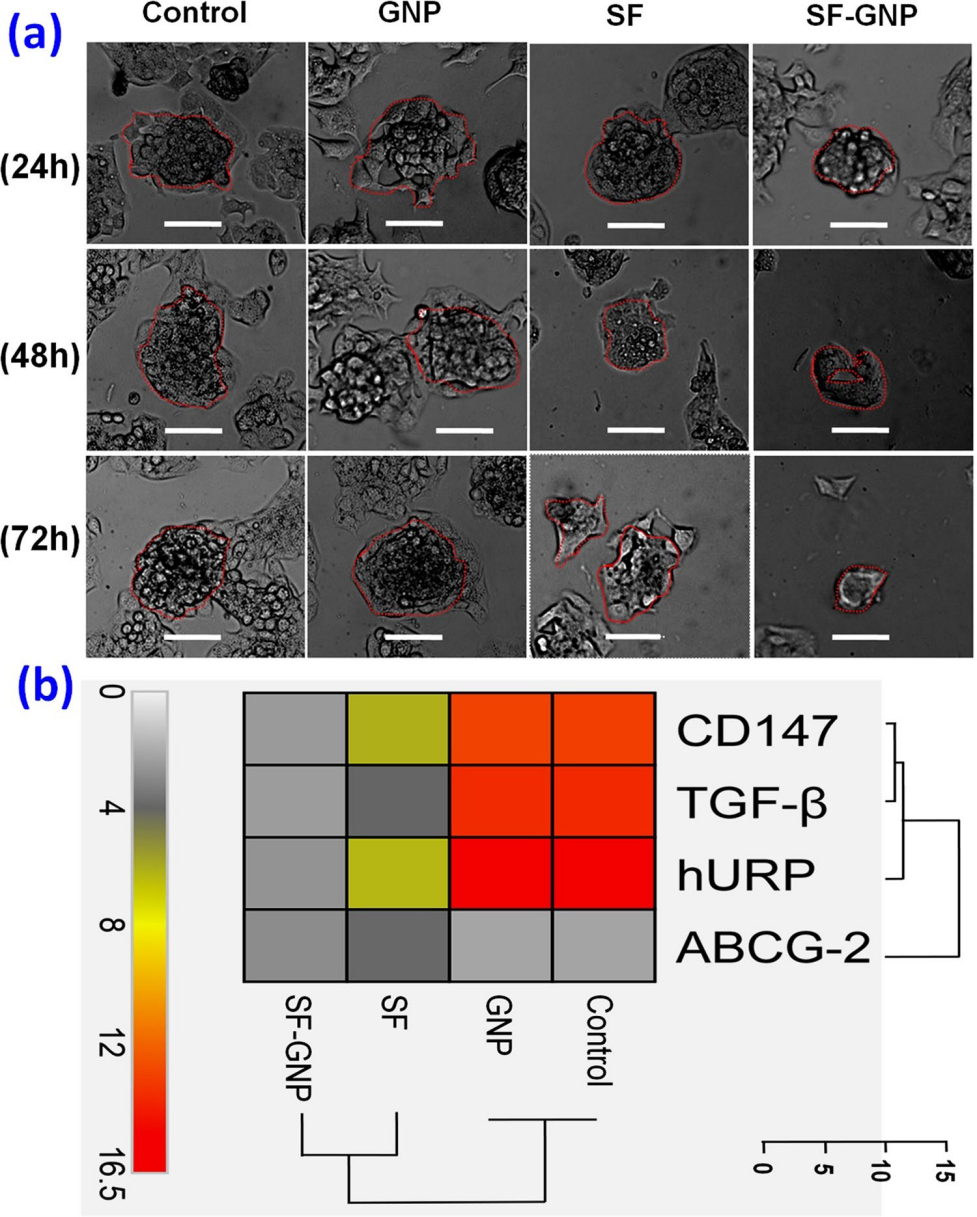
(**a**) The developing HepG2 cell colonies showed softening of tumor colonies with loosely arranged cells after SF-GNP treatments as compare to other treatment conditions. (Scale bar: 20 µm, magnification: 40X). (**b**) Heat map with clustering algorithm analysis showing the expression pattern of cancer markers (hURP, CD147 and TGF-β) and drug transporter (ABCG2) transcripts at day 3 post treatments of growing HepG2 cell colonies.

The effect of SF-GNP nanoconjugates was identified by analyzing the size and number of solid tumor HepG2 cell colonies in semi-solid agar medium which showed gradual decrease in colony number (Fig. 4d–f) and size (Fig. 4g–i) at day 1, day 2 and day 3 respectively. The average size of solid tumor colonies significantly reduced more than 80% after treatment with SF-GNP nanoconjugate whereas the average size for free SF was found increased by two fold as compared to the SF-GNP nanoconjugate treated colonies (Fig. 5a). The tumor developing colonies revealed softening of tumor colonies without proper assimilation of cells within the colonies in SF-GNP treated groups as compared to others. Further the molecular basis of anti-proliferative effect of SF-GNP nanoconjugate was identified by relative quantification of extracellular matrix factor (CD147), tumor growth factor (TGF-β), hepatoma upregulated protein (hURP) and drug transporter (ABCG-2) transcripts. The transcript quantification showed significant decrease in expression at day 3 post treatment with SF-GNP as compared to free SF, GNPs and control (Fig. 5b). The clustering algorithm of the transcript expression showed distinct characteristic homology between control with GNPs and free SF with SF-GNP nanoconjugate. Elucidation distance for the transcripts expression in different groups showed distinct down regulated expression with higher elucidation distance for ABCG2 as compared to CD147, TGF-β and hURP.

### SF-GNP nanoconjugates to combat SF resistant HepG2 cells

**Figure 6:**
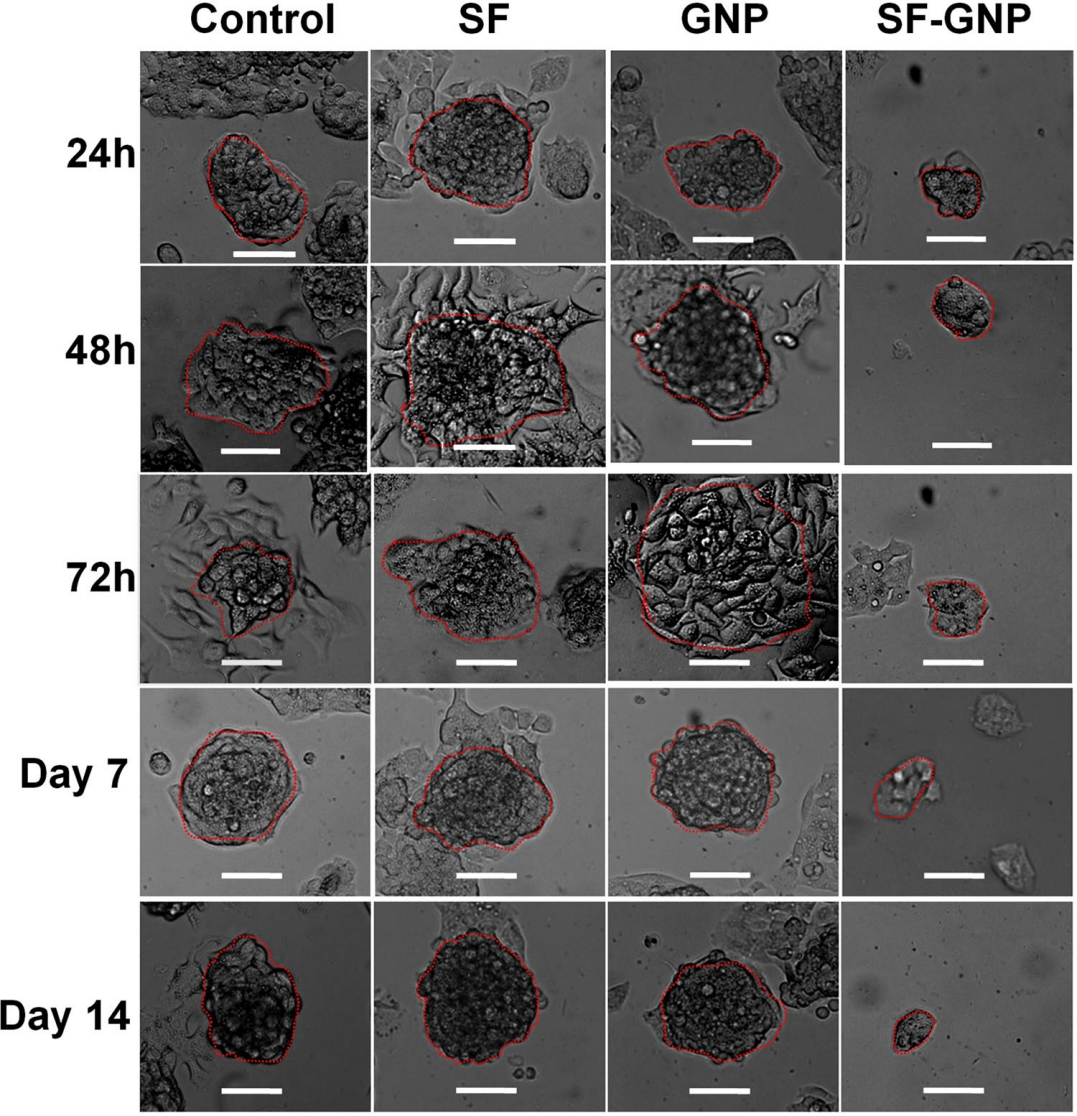
Treatment with SF-GNP conjugate reduces the HepG2 cell survival and enhances the colony deformalities with alterations in transcriptional activation in SF resistant hepG2 cells in 3D culture system. (**a**) Microscopic analysis during long-term exposure of SF-GNP in growing HepG2 cell colonies showed reduction in colony size and cellular packaging with increasing the time of exposure. (Scale bar: 20 µm, magnification: 40X).

**Figure 7:**
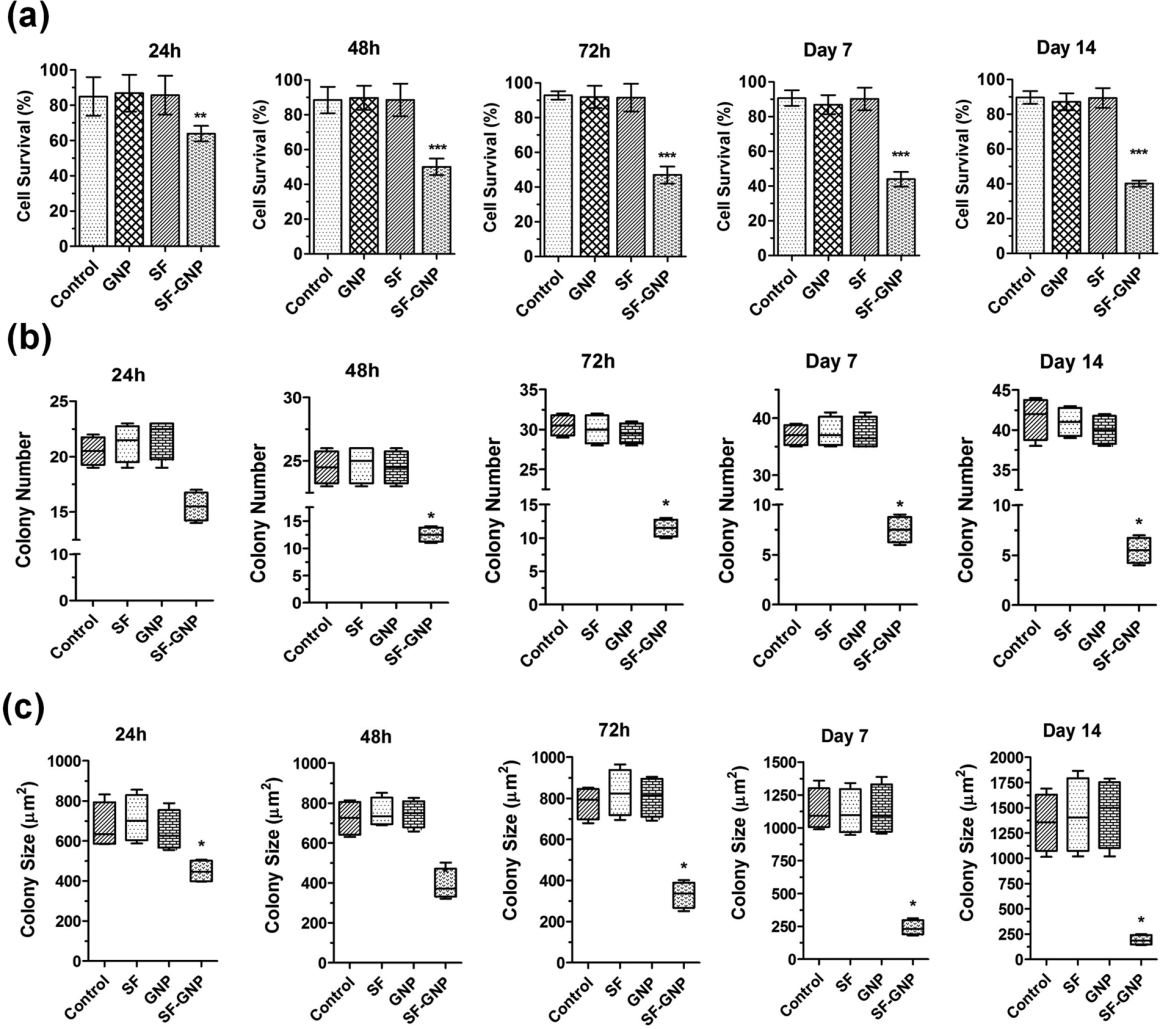
The percentage cell survival was found to be significantly decreased from day 1 to day 14 in colonies treated with SF-GNP conjugate as compared to free SF (40% at day 14, p < 0.0001). (**c**) Reduction in colony number was gradually increased with progression of time for SF-GNP treatment and was significantly high at day 14 (approximately 20%, p < 0.01). (**d**) Further the number of SF resistant HepG2 cell colonies was significantly declined with the time and was significantly less at day 14 (approximately 14%, p < 0.01) of SF-GNP treatment duration.

To evaluate the effect of SF-GNP nanoconjugates on SF resistant HepG2 cells, 12-well culture plates were used for initial development of SF resistant HepG2 colonies for 14 days with similar survival rate. After 14 days, the colonies were exposed to free SF, GNP and SF-GNP nanoconjugate and cell viability was assessed using mitochondrial dehydrogenase activity and FDA flow cytometry analysis. The percentage cell survival was decreased significantly from day1 to day 14 in colonies treated with SF-GNP nanoconjugates as compared to free SF (Figs 6 and 7a). The flow cytometry analysis also revealed similar pattern with MTT data of reduced cell survival of ~50% at day 3 and ~40% at day 14 (Fig. S4.2). The colony number was reduced up to 90% of SF resistant colonies at day 14 post exposure to SF-GNP (Fig. 7b). Similarly, the colony size was also reduced gradually and was found to be approximately 14% of initial size at day 14 post SF-GNP treatment (Figs 6 and 7c).

**Figure 8:**
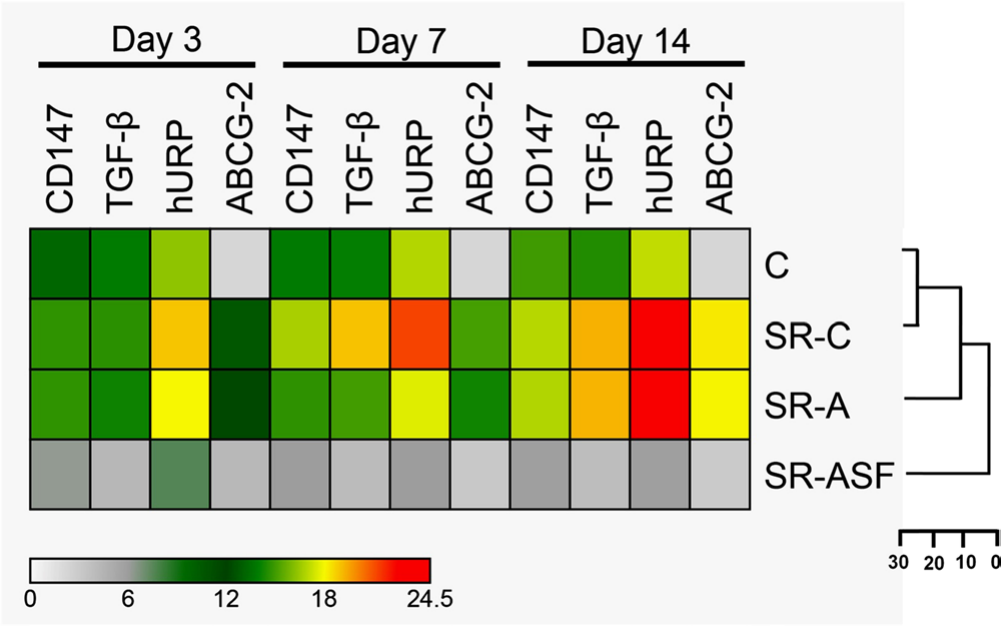
Heat map showing relative quantification of major cancer pathway transcripts (hURP, TGF-β), Warburg oncogene (CD147) and MDR transcript ABCG2 at day 3, 7 and 14 post-treatment of SF resistant HepG2 cell colonies with SF-GNP nanoconjugate. The analysis showed significant down-regulation for all the transcripts after treatment with SF-GNP with increasing the time of treatment (p < 0.0001). Highly reduced expression of ABCG2 in SF-GNP treated SF resistant colonies (p < 0.0001) provides evidence for the inhibition of drug efflux mechanisms which prevents the drug uptake.

We also sought to elucidate the underlying mechanisms of drug resistance in HepG2 cells in solid HCC model system by investigating the implications of hURP^16^, CD147/Basigin^17^, TGF-β^18, 19^ and ABCG2^20^. The transcript expression analysis revealed no significant change in SF resistant cells derived from respective colonies treated with free SF. However, significant down-regulation for all the transcripts was observed in SF resistant HepG2 cells derived from respective colonies after treatment with SF-GNP nanoconjugate (*p* < 0.0001, Fig. 8). Highly reduced expression of ABCG2 in SF-GNP treated SF resistant HepG2 cell colonies (*p* < 0.0001) provides evidence for the inhibition of drug efflux mechanisms which prevents the drug uptake. It  was further validated by western blot analysis of ABCG-2 protein level in SF resistant HepG2 cell colonies at day 14 (Fig. S6.3) post-treatment with SF-GNP nanoconjugates. Correlation between data obtained from all four different groups (control, SF, GNP and SF-GNP) showed completely different reduction pattern in ABCG2 expression in SF- GNP group as compared to others. This was further validated by elucidation distance analysis in heat map showing larger distance and distinct sub-family within the expression pattern for drug transporter.

## Discussion

The major focus of using GNPs in cancer has been due to its unique multifaceted properties and size dependent EPR effect^5^. Higher EPR effect in cellular systems can be achieved by using small size (1–100 nm) nanoparticle-based drug delivery^5, 21^. Here the effective targeting and increase in drug uptake is expected due to small size (<10 nm) of SF-GNP conjugates which is at the lower side of the 1–100 nm reported range of higher EPR effect. Additionally, the molecular functionality of SF in SF-GNP nanoconjugate can also support active targeting to further enhance effective delivery^22^. The active targeting of SF-GNP is due to multi-targeted effect of SF in cancer cells. So, along with passive targeting through diffusion and accumulation in cancer cells, SF-GNP nanoconjugate will also affect the cancer cells through binding on cancer cell surface receptors such as Raf, VEGF and PDGF. The improved response of SF-GNP conjugates observed in SF resistant HepG2 cell colonies provides a new dimension in the treatment of drug resistant cancer cells.

Previously, different types of GNPs with various functionalities were used for enhanced drug transportation in cancer cells^23–25^. The cytotoxicity of citrate capped GNPs explored as self-therapeutic nanoparticles for inhibiting of tumor growth and metastasis^26^. Previously doxorubicin-tethered responsive GNP used for intracellular drug delivery in MDR MCF-7/ADR cancer cells^27^. The use of PEGylated GNPs for preparing conjugates with doxorubicin showed increased drug uptakes in MDR cancer cells^28^. GNPs conjugated with β-cyclodextrin enclosing paclitaxel were effectively endocytosed by both drug-sensitive human lung cancer cells^29^. These studies confirms the usefulness of small size nanoconjugates for effective diffusion but their biocompatibility has not been well studied. Thus, in our view, this is the first report identifying the enhanced therapeutic value of biocompatible SF-GNP nanoconjugate in drug resistant HepG2 cells.

During exposure, nanoparticles/drugs are transported through blood and reach the systemic circulation from where they distribute and accumulate into several vital organs which further confirms the nanoparticles design to overcome on biological barriers in drug delivery. Kidney and hepatobiliary systems are two major components responsible for the elimination and clearance of foreign materials. Other organs like lung and heart are primary sites of nanoparticles accumulation and can show early response for positive or negative impact. The brain is restricted for direct entry of nanoparticles due to blood brain barrier, however nanoparticles impact on brain cells have also been observed which could be through cerebrospinal fluid. Therefore, in present study, histopathological analysis of liver, brain, lung, kidney and heart tissues were performed following IP administration of GNP, SF and SF-GNP at day 3 and day 14 post exposure. The motivation for IP administration of SF-GNP nanoconjugate was that the peritoneum is highly rich vascularized immunologically privileged site, it does not produce higher immunological response immediately due to absence of large number of immunological cells as compared to the other sites. In addition, these nanoconjugates are very small (<10 nm), hence can easily enter through endothelial barrier of the blood vessel in peritoneum (>100 nm in size) which leads their direct accumulation into the liver through gastro-intestinal tract. This site also has advantages over rescue of the reticulo-endothelial uptake by immunological cells which is a positive sign for enhanced delivery at the targeted cells. This investigation was carried out to uncover and understand the tissue toxicity and predict their potential therapeutic values. Herein, the synthesis of non-functionalized GNPs in aqueous medium has potential to overcome the limitations of earlier reports of GNPs toxicity and provides safer nanoscale system to treat drug resistant solid tumor cells. The *in-vivo* cytotoxic effect of SF-GNP nanoconjugate in blood clearly shows that SF-GNP conjugate does not induce interference in hematological parameters without hemolysis (Tables S2.1–2.3). Additionally the strength of biocompatibility for SF-GNP conjugate was confirmed by observing normal range of liver and renal functions similar to controls (Fig. S2.4). The histopathological analysis of vital organs (liver, brain, lung, kidney and heart tissues) confirmed the absence of inflammatory cell infiltration and tissue damage due to the systemic distribution and accumulation of SF-GNP through circulation^30^. More specifically normal structural organization, absence of tissue necrosis and fibrotic reactions or cell infiltration revealed the biocompatibility and least systemic adverse response in vital organs against GNP and SF-GNP. More detailed investigation with the help of veterinary pathologist in blinded manner the histopathologcial score were predicted using atleast 10 areas of one section of each type of tissue stained with hematoxylin and eosin. No signifcant change in scoring pattern during GNP or SF-GNP nanoconjugate exposure revealed their biologically safe nature for systemic applicability (SFig. 2.5). Conclusively, the biocompatibility results presented herein for different *in-vivo* serum, blood and tissue analysis encourages for the safe applicability of SF-GNP conjugate in biological system.

This particular strategy of using SF-GNP conjugate in treating SF resistant HCC cells may augment the possibility of SF and related drugs to reduce the load of drug resistance with lower dose and reduced adverse events in advanced cancer by evading the drug efflux mechanisms^31^. At this stage, we do not understand the mechanism by which SF-GNP nanoconjugates are interacting with DR-HepG2 and the effect of acidic environment on stability of SF-GNP nanoconjugates within the cellular system. Further studies will be required to better understand the internalization and subsequent release of SF from SF-GNP nanoconjugates. In addition, the long term therapeutic efficacy of SF-GNPs in preclinical model system with detailed mechanism for uptake of SF-GNP nanoconjugates and reversal of drug resistance needs to be identified.

## Methods

### Materials

Gold Chloride (AuCl_3_), Lithium borohydride (LiBH_4_) and Fluorescent isothiocayanate (FITC) were purchased from Aldrich-Sigma. All the experiments were performed in Milli-Q water (Merck- millipore, USA).

### Preparation and fluorometric assay of FITC functionalized GNPs

Gold nanoparticles (GNPs) were synthesized by instantaneous reduction of 0.2 mM of AuCl_3_ with 6 mM of LiBH_4_ by adding freshly prepared AuCl_3_ with LiBH_4_. FITC functionalized GNPs were prepared by adding 4 µl of 0.5 mM FITC (in 99.9% ethyl alcohol) in 3 ml of GNPs colloidal suspension. Fluorescence spectra of FITC-GNP colloidal suspension were obtained after incubating for 10 min at room temperature and characteristic emission peak between 500–650 nm was observed for FITC solution at 478 nm excitation.

### Sorafenib Gold Nano-conjugate

Sorafenib solution was prepared by taking 285 mg of SF tablet powder dispersed in 40 ml of water by sonicating it for 15 min. Further it was diluted to make a solution of ∼0.5 mg/mL SF as a stock. For preparation of SF-GNP Nanoconjugates (**SF-GNP**), 2 to 1 volume to volume ratio of GNP and SF stock solution mixed well by sonicating for 5 mins and left at 4 °C for 72 hr. Quantitative estimation of the amount of SF binds on GNP was performed by HPLC by comparing the HPLC chromatograms of SF solutions prepared in water and similar concentration of SF-GNP nanoconjugates.

### Animal Groupings and Treatments

Male Wistar Rats of average body weight 140 ± 25 g which were obtained from animal house after obtaining ethical clearance from institutional animal ethics committee of Centre for Cellular and Molecular Biology (CCMB), Hyderabad as per the Indian Council of Medical Research (ICMR) guidelines. Animals were placed in clean environment and allowed to acclimatize for a week before start of treatments. Animals were given commercial mice chows and clean water was supplied. Animals were randomly assigned into different categories of four rats per group. Treatment was carried out with three different types of drug formulations (i) The GNPs diluted in distilled water in 2:1 ration (**GNP**), (ii) **SF** and (iii) 0.33 mg/mL SF with 2:1 ratio diluted with GNP (**SF-GNP**). Animals in each category received (2 µL/gm weight of animal) intraperitoneal (IP) injection of (G1) water as control standard (**Control**), (G2) GNP treatment (**GNP**), (G3) SF treatment (**SF**) and (G4) SF with GNP treatment (**SF-GNP**). Treatments were intraperitonially (**IP**) administered each day for first 3 days. Details of blood parameter, serum and histological analysis of different organs are given in supporting information.

### *In vitro* cell culture

The human hepatoblastoma cell line (HepG2) was cultured in T-25 flasks in Dulbecco minimal essential medium (DMEM-F12, Gibco) supplemented with 10% fetal bovine serum (FBS), 2 mM L-glutamine, 4.5 gm/L glucose, and 1X antibiotic solution. The cells were maintained under controlled conditions of 5% CO_2_ and 37 °C temperature in a CO_2_ incubator (Thermo scientific). The culture medium was changed every 2^nd^ day by replenishing 50% of fresh medium. Cell confluency was observed by optical microscopic observation of cells in culture and tripsinized before reaching to 80% confluency. Cells were maintained for 10 passages and passage 4 and 5 cells were used for further experimentation. Details of different analysis given in supporting information.

### Development of SF resistance in HepG2 cells

The HepG2 cells were cultured and IC_50_ values of SF from dose response curve optimized. The IC_50_ of SF in sensitive HepG2 cells was observed 17.5 µg/mL among different range of concentrations tested. The SF resistant cells were developed by increasing concentration 25% each time of SF above 17.5 µg/mL (i. e. 21.87 µg/mL to 100 µg/mL). Finally the SF resistant HepG2 cells were found to grow exponentially at 52.48 µg/mL concentration of SF which was designated as the optimum concentration of SF to develop resistant HepG2 cells (SF-DR). The IC_50_ value of this concentration of SF was calculated by 52.48/2 which was found to be 26.24 µg/mL and denoted as IC_50_ of SF in SF-DR HepG2 cells.

Further, the resistance index (RI) of SF was determined as follows: RI of SF = IC_50_ of SF resistant (SF-DR) HepG2 cells/IC_50_ of sensitive HepG2 cells. Thus RI = 26.24/17.5 giving the value of 1.49. Similarly, the IC_50_ of SF-GNP in sensitive HepG2 cells was determined to be 5.8 µg/mL whereas in resistant HepG2 cells the IC_50_ was 6.264. Hence, the resistance index (RI) of SF-GNP in resistant cells was determined as follows: RI of SF-GNP = IC_50_ of resistant HepG2 cells/IC_50_ of sensitive HepG2 cells which is equal to 6.264/5.8 and giving a final value of 1.08. Hence, 26.24 µg/mL (IC_50_) concentration of SF was used to treat HepG2 cells regularly for seven passages each for three days. The cell viability was determined by MTT assay, at each passage of day 3 after SF treatment. The SF treated HepG2 cells passage with viability of approximately 100% or similar to control group was considered as SF resistant cells which was further utilized to determine the SF-GNP therapeutic efficacy in 3D solid HCC tumor model system.

### Establishment of *ex vivo* 3D HCC model system

We established an *ex vivo* 3D HCC model by using modified protocol of soft agar colony formation assay due to the better correlation between *in vitro* transformation and *in-vivo* carcinogenesis. This method provides more stringent approach for evaluating the tumorigenic response and tumor suppressive effects of drugs on cancer cells. Briefly, the base agar layer was prepared by adding one milliliter of 0.75% low melting agar prepared in culture medium in each well of 12 well culture plates and allowed to solidify for 10–20 min at room temperature. After solidification of base agar, a top agar layer was prepared by adding one milliliter of 0.36% low melting agar in culture medium along with cells (10^5^ per well) and allowed to solidify at room temperature. Additional one milliliter of cell culture medium was added into each well and incubated in CO_2_ incubator at 37 °C for two weeks before performing the toxicity experiments. The medium was replenished every 3^rd^ day and the volume of growing colonies was observed by phase contrast microscopy (Carl Zeiss, Germany). For establishing the drug resistant 3D HCC model system, SF-resistant HepG2 cells from passage 6 were used to develop SF resistant colonies for screening the effect of SF-GNP conjugate in time and dose dependent manner.

### Estimation of percentage cell survival and growth statistics in 3D HepG2 and SF resistant HepG2 culture model system

Percentage cell survival in developing HepG2 colonies post treatment with GNP, free SF and SF-GNP conjugate was measured with FDA flow cytometry and MTT cell viability assays. In brief, agarose solubilization buffer was used to dissolve the agarose and cells were harvested from the colonies, trypsinized and stained with FDA (2 mg/mL) for 5 min at room temperature followed by two time wash with 1X PBS. FDA stained cells were subjected to flow cytometry analysis using Cell Quest software. The results obtained from flow cytometry analysis were further validated by MTT cell viability assay. Herein, after dissolving the agarose with solubilzation buffer, 100 µL of the mixture was transferred to a 96-well microtiter plate in each well and 10 µL MTT was added and mixed 6–8 times to ensure the homogeneous mixing and incubated for 4 h in CO_2_ incubator. These experiments were performed after completion of the time for treatment. The reaction was stopped by adding 100 µL DMSO in each well and further incubated for 10 min. After the incubation, absorbance was measured by microplate reader at 570 nm which was correlated with the controls and FDA flow cytometry data. These procedures were followed for both the non-drug resistant HepG2 and SF resistant HepG2 cells during time and dose dependent toxicological studies of SF-GNP conjugate. The anchorage dependent cell growth was calculated by following formula: Total transformed cells/well = cells/mL × 0.050 mL/well.

### Determination of colony number and size

Colony number was determined microscopically by manual colony counting in triplicate wells of each group. Further to facilitate the quantification process, a grid was printed onto a transparency and attached to the 12 well plates to locate the colonies during counting. Whereas, due to variability in colony size (quantified as diameter of each colony), the average size of colonies in each well was calculated and compared with other groups. To measure the colony size, Axiovert software was applied using phase contrast microscopy (Carl Zeiss, Germany).

### Gene expression analysis

Total ribonucleic acid (RNA) was extracted from HepG2 cells after dissolving agarose in solubilization buffer before and after treatment with SF-GNP in normal and SF resistant HepG2 cells using standard GITC method. Complementary de-oxy ribonucleic acid (cDNA) was prepared using total RNA extracted from each group with the help of Oligo dT primers (Invitrogen) and reverse transciptase enzyme-II (Fermentas, Canada). cDNA was further quantified using nanodrop reading and 5ng cDNA was used for gene expression analysis. SYBR Green-based relative quantification of hURP, CD147, TGF-β and ABCG2 was performed in StepOne RT-qPCR (Applied Biosystem, USA). Glyceraldehyde 3-phosphate dehydrogenase (GAPDH) was used for the normalization of test samples against endogenous contro (GAPDH). PCR efficiency of each transcript was calculated by using Y = mx + c formula. Each transcript was analyzed two times in triplicates in three different set of experiments. The mean was taken for each sample and the relative fold value of each transcript was calculated using the 2^−ΔΔCT^ method by StepOne (Version 2.2) software in StepOne Real-Time PCR.

### Statistical analysis

The data was represented as mean ± SEM. One way and two way ANOVA was performed using Graph Prism software for variance analysis and comparison of different groups. R programming was used for statistical computing and heat map representation of fold difference values for different mRNA transcripts. The data were compiled and run on a UNIX platform during R programming (R Version 3.1.2) to create the heat map and estimate the elucidation distance among different groups. A *p* value less than 0.05 was considered statistically significant.

All other experimental details are provided in supporting information.

## Electronic supplementary material

Supporting Information
