# Supplementary material for: Use of Biocompatible Sorafenib-gold Nanoconjugates for Reversal of Drug Resistance in Human Hepatoblatoma Cells
Source: Sci Rep. 2017 Aug 17;7:8539. doi: 10.1038/s41598-017-08878-y (PMC5561190; doi:10.1038/s41598-017-08878-y)
Supplement: Supplementary file 1 — Supporting Information [file 41598_2017_8878_MOESM1_ESM.doc]

**Supporting Information**

**Use of Biocompatible Sorafenib-gold Nanoconjugates for Reversal of Drug Resistance in Human Hepatoblatoma Cells**

*Sandeep Kumar Vishwakarma^1,2^, Priyanka Sharmila^1^, Avinash Bardia^2^, Lakkireddy Chandrakala^2^, N. Raju^2^, Sravani G^2^, B.V.S. Sastry^2^, Md. Aejaz Habeeb^2^, Aleem Ahmed Khan^2*^, Marshal Dhayal^1,3*^*

^1^Clinical Research Facility, Medical Biotechnology Complex, CSIR-Centre for Cellular and Molecular Biology, Uppal Road, Habsiguda, Hyderabad 500007, Telangana, India.

^2^Central Laboratory for Stem Cell Research and Translational Medicine, Centre for Liver Research and Diagnostics, Deccan College of Medical Sciences, Hyderabad 500058, Telangana, India.

^3^School of Biomedical Engineering, Indian Institute of Technology (Banaras Hindu University), Varanasi – 221005, India.

*Corresponding author (marshaldhayal@yahoo.com (MD) and aleem_a_khan@rediffmail.com (AAK), phone: +91-(0)9652013844, fax: +91-(0)-542-2368428)

**Different types of analysis were performed as follows:**

***Blood collection and Serum Separation***

Blood was collected on Days 3, day 7 and day 14 from the orbital sinus using isoflurane drop jar. After the blood was collected it was incubated at 27±2°C for 2 hours and centrifuged at 3000 xg for 15 min at 4˚C. Finally the serum as supernatant was collected. The collected serum was stored at -20˚C for further analysis.

***Analysis of serum biochemical parameters***

Whole blood collected from each group of animals was centrifuged at 3000 rpm for 10 min to separate the serum. Biochemical analyzer (Microlab 200, Merck) was used for the analysis of serum biochemical parameters to quantify the serum levels of liver function parameters such as alkaline phosphatase (ALP, BLT0003, Erba lachema, CZ), and alanine amino transaminase (ALT/SGPT, BLT00052, Erba lachema, CZ), aspartate transamino-transferase (AST/SGOT, BLT00050, Erba lachema, CZ), total bilirubin (BLT00011, Erba lachema, CZ) and albumin (BLT00001, Erba lachema, CZ) and renal function parameters such as urea (BLT00060, Erba lachema, CZ) and creatinine (XSYS0024, Erba lachema, CZ).The biochemical analysis of the serum levels of above parameters was analyzed by standard laboratory procedures by using their respective kits as per the manufacturer’s instructions.

***Assessment of hematological parameters***

Blood samples were drawn in EDTA-coated tubes for hematology analysis. The samples from each group in triplicate were run on hematological autoanalyzer (Pentra ES_60_, Horiba, France) to determine complete blood count, including red blood cells (RBC), white blood cells (WBCs), hemoglobin (HGB), neutrophils, basophils, eosinophils, monocytes, lymphocytes, hematocrit (HTC) and platelets (PLT). The values were recorded and mean±SD was calculated to find the variation among GNP, free SF and SF-GNP groups at post-injection day 3, day 7 and day 14.

**Histological investigations**

The inflammation and damage in liver tissue was performed by histological analysis using Knodell score or histologic activity index as described elsewhere (**Batts et al. 1995; Brunt et al. 2009**). Score 0 was given for the complete absence of the liver inflammation, 1-4 for minimal, 5-8 for mild, 9-12 for moderate and 13-18 for marked severe inflammation. Similarly renal inflammation and histopathological analysis was performed by a veterinary pathologist. The scoring was performed after carefully analysis of renal glomerular degeneration and renal tubule necrosis as described recently by **Gu et al. (2016)**. To score the lung tissue inflammation, the lung sections affected by inflammation and/or fibrosis were scored semi-quantitatively by using modified protocols (**Matute-Bello et al. 2011**). The total “lung inflammations score” was predicted as sum of the total score of each parameter. Damaged and/or inflamed cardiac tissues were scored by histopathological analysis as per the standard scoring method (**Hölschermann et al. 1999**). The percentage of damage in brain tissues was evaluated in different areas of the brain and expressed as percentage damage as described earlier (**Sarah et al. 2014**).

**References**

- Elizabeth M. Brunt, David E. Kleiner, Laura A. Wilson, Aynur Unalp, Cynthia E. Behling, Joel E. Lavine, Brent A. Neuschwander-Tetri, and the NASH CRN. Portal Chronic Inflammation in Nonalcoholic Fatty Liver Disease: An Histologic Marker of Advanced NAFLD Clinicopathologic Correlations from the NASH Clinical Research Network. Hepatology. 2009 March ; 49(3): 809–820. doi:10.1002/hep.22724.
- Batts KP, Ludwig J. Chronic hepatitis. An update on terminology and reporting. Am J Surg Pathol. 1995;19:1409-17.
- Sing-YiGu, Ti-YenYeh, Shih-Yi Lin & Fu-Chuo Peng. Unfractionated bone marrow cells attenuate paraquat-induced glomerular injury and acute renal failure by modulating the inflammatory response. Scientific Reports, 2016; 6:23287 | DOI: 10.1038/srep23287.
- Matute-Bello G, Downey G, Moore BB, Groshong SD, Matthay MA, Slutsky AS, Kuebler WM; Acute Lung Injury in Animals Study. An official American Thoracic Society workshop report: features and measurements of experimental acute lung injury in animals. Am J Respir Cell Mol Biol 44: 725–738, 2011.
- Hans Hölschermann, Rainer M. Bohle, Hagen Zeller, Heiko Schmidt, Ulrich Stahl, Ludger Fink, Helmut Grimm, Harald Tillmanns, Werner Haberbosch. In Situ Detection of Tissue Factor within the Coronary Intima in Rat Cardiac Allograft Vasculopathy. American Journal of Pathology, January 1999; Volume 154, Issue 1, Pages 211–220.
- Sarah B. Simmons, Denny Liggitt and Joan M. Cytokine-Regulated Neutrophil Recruitment Is Required for Brain but Not Spinal Cord Inflammation during Experimental Autoimmune Encephalomyelitis. J Immunol July 15, 2014, 193 (2) 555-563; DOI: https://doi.org/10.4049/jimmunol.1400807

***Determination of IC_50_ value for SF using dose response curves analysis***

To determine the IC_50_ value of SF, a dose dependent experiment was performed in HepG2 cells using cell proliferation assay. The cells were incubated for 72h with respective doses of SF. After 72h of exposure, MTT (3-(4,5-dimethylthiazol-2-yl)-2,5-diphenyltetrazolium bromide) cell proliferation assay was performed to identify the cell death. The concentration responsible for killing of 50% HepG2 cells was noted and considered as IC_50_ value of SF and utilized for further experimental purposes.

***Determination of colony number and size***

Colony number was determined microscopically by manual colony counting in triplicate wells of each group. Further to facilitate the quantification process, a grid was printed onto a transparency and attached to the 12 well plates to locate the colonies during counting. Whereas, due to variability in colony size (quantified as diameter of each colony), the average size of colonies in each well was calculated and compared with other groups. To measure the colony size, Axiovert software was applied using phase contrast microscopy (Carl Zeiss, Germany).

***Dose dependent experiment on SF resistant HepG2 cell colonies***

We have developed SF resistance in the human hepatoblastoma cell line (HepG2) which was cultured and IC_50_ values of SF from dose response curve optimized (see supporting information for more details), 17.5 µg/mL (IC_50_) concentration of SF was used to treat HepG2 cells regularly for seven passages each for three days. The cell viability was determined by MTT assay, at each passage of day 3 after SF treatment. The SF treated HepG2 cells passage with viability of approximately 100% or similar to control group was considered as SF resistant cells which was further utilized to determine the SF-GNP therapeutic efficacy in 3D solid HCC tumor model system. Further, to demonstrate the usefulness of the SF-GNP nanoconjugates for the treatment of drug resistance cancer, an *ex-vivo* 3D HCC model for time and dose dependent high throughput cellular toxicity and genetic studies was established by using modified protocol of soft agar colony formation assay described elsewhere [20,21]. This model system enables us to have a better correlation between *in-vitro* transformation and *in-vivo* carcinogenesis.

To determine the effect of different doses of SF-GNP conjugate on SF resistant HepG2 cells in solid tumor model system, single, double and triple dose experiment was conducted for SF-GNP to identify the percentage cell survival and colony development at day 3, 7 and 14 post treatments. Further to evaluate the effect of different concentrations of SF-GNP, two additional dilutions were made (1/10 and 1/100) and used to expose SF resistant HepG2 cell colonies for day 3, day 7 and day 14 at single, double and triple dose of each concentration.

***Gene expression analysis***

*Primers sequences used for quantitative RT-PCR*

Hepatoma upregulated protein (hURP), forward: AAGCCTCGTTGAGTGGAAGG, reverse:AAGCAGGAACCCTCACAACC, CD147/BSG, forward: AGTGTAGCCACATTCCTGCC, reverse: GGCGTCTCTTGGAGGTTGAA, TGF-β, forward: TCCATTGACGACGCCTTGG, reverse: CCCTCTCTAACGTCTTGAGTCT, ABCG2, forward: GTTCTCAGCAGCTCTTCGGCTT, reverse: TCCTCCAGACACACCACGGATA, GAPDH, forward: GTCTCCTCTGACTTCAACAGCG, reverse: ACCACCCTGTTGCTGTAGCCAA.

*mRNA isolation*

Total ribonucleic acid (RNA) was extracted from HepG2 cells after dissolving agarose in solubilization buffer before and after treatment with SF-GNP in normal and SF resistant HepG2 cells using standard GITC method as described earlier [15]. RNA was quantified and purity was calculated by nanodrop reading (Thermo scientific). The ratio of absorbance at 260 and 280nm was used to assess the purity of RNA and the ratio of ~2.0 was considered as highly pure RNA.

*cDNA construction*

Complementary de-oxy ribonucleic acid (cDNA) was prepared using total RNA extracted from each group with the help of Oligo dT primers (Invitrogen) and reverse transciptase enzyme-II (Fermentas, Canada). cDNA was further quantified using nanodrop reading and 5ng cDNA was used for gene expression analysis.

*Relative quantification*

SYBR Green-based relative quantification of hURP, CD147, TGF-β and ABCG2 was performed in StepOne RT-qPCR (Applied Biosystem, USA). Glyceraldehyde 3-phosphate dehydrogenase (GAPDH) was used for the normalization of test samples against endogenous contro (GAPDH). PCR efficiency of each transcript was calculated by using Y = mx + c formula. Each transcript was analyzed two times in triplicates in three different set of experiments. The mean was taken for each sample and the relative fold value of each transcript was calculated using the 2^-ΔΔCT^ method [16] by StepOne (Version 2.2) software in StepOne Real-Time PCR.

**Instruments**

The double beam Perkin Elmer Lambda 35 Spectrophotometer was used for UV-Visible absorption spectra. Fluorescence emission spectra were obtained with Hitachi 4500 Japan spectrophotometer having slit width of 5nm. Excitation and emission wavelengths of FITC were used at 488nm and 520nm. The TEM micrographs of AuNPs and modified AuNPs with SF and FITC were obtained by 200KV Transmission Electron Microscope, from JEOL, Japan. The median value and S.D was considered for plotting the graphs were obtained from the measurements which were conducted for minimum three times in triplicates.

SF detection was carried out in an Agilent 1260 infinity HPLC system consisting of a Quaternary pump and a UV–Vis detector. Analysis was carried out on a Zorbax C18 column (250×4.6 mm, 5μm). We have used time gradient programming in which the composition of the mobile phase at time zero (the time of injection) was 40% ammonium acetate (20mM) and 60% acetonitrile. The percentage of acetonitrile was then increased to 72% over 9 min. Then, the composition was changed back to ammonium acetate-acetonitrile (40:60; v/v) within 6s. Finally, the chromatographic system was equilibrated during 5min before the next injection. The flow rate was 1ml/min and temperature 40°C throughout the 14min run time. The detection wavelength was set 255 nm for SF which was about 5 nm less than the SF peak position. The diluent was prepared by mixing HPLC grade Acetonotrile: 20mM ammonium acetate (60:40). The resultant solution of the diluent was filtered through 0.45μ membrane filter.50 μl of different concentrations (1μg/mL-1mg/mL) of SF solutions were injected and the chromatograms were recorded.

**
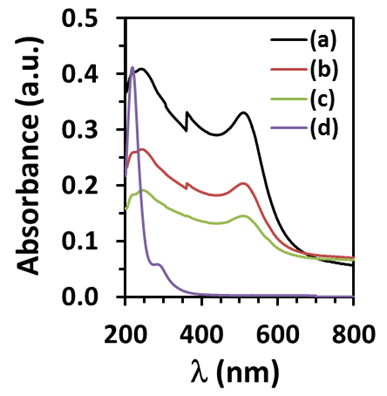
**

**Fig. S1.1:** UV-vis spectra of GNP at various dilutions in water. (a) as synthesized, (b) ½ dilution, (c) ¼ dilution and (d) gold chloride salt in water.


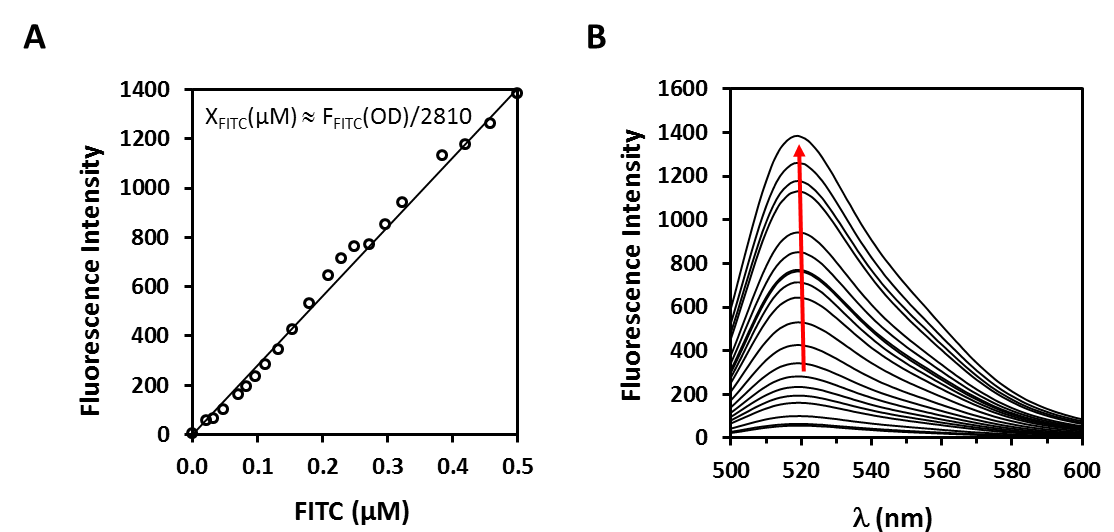


**Fig. S1.2:** Linear response of FITC in water. (A) FITC fluorescence peak intensity at 520nm in fluorescence spectra at various concentrations of FITC in water and (B) corresponding fluorescence spectra (arrow indicates an increase in FITC concentrations).


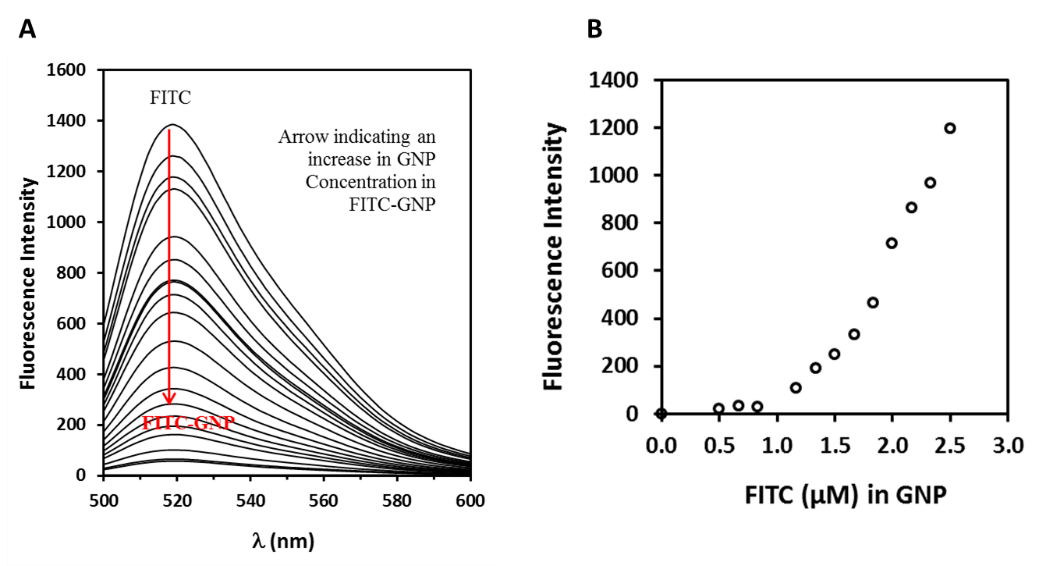


**Fig. S1.3:** Fluorescence quenching of FITC with GNP. (A) Fluorescence quenching of FITC in FITC-GNP at various concentrations of GNP (B) FITC fluorescence peak intensity at 520nm in fluorescence spectra at various concentrations of FITC in GNP.


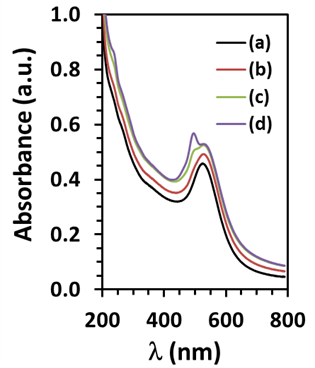


**Fig. S1.4:** UV-vis spectra of GNP at various levels of FITC functionalization (a) GNP, (b) 0.66 µM FITC in 3 mL of GNP, (c) 1.33 µM FITC in 3 mL of GNP, and (d) 2.66 µM FITC in 3 mL of GNP.


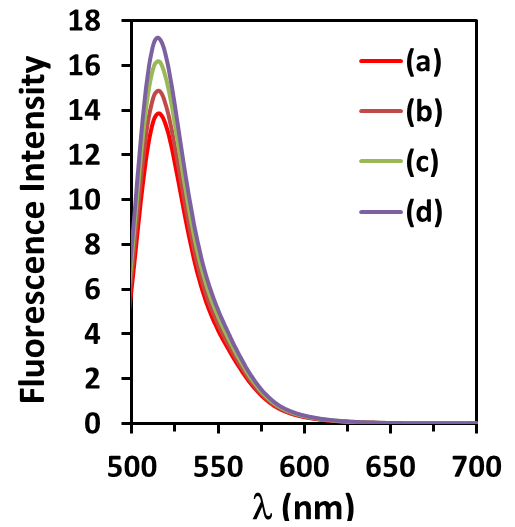


**Fig. S1.5:** Fluorescence spectra of FITC released from FRET FITC-GNP nanprobe at various concentrations of SF (a) 4 µL of SF (1mg/mL) in 0.66 µM FITC in 3 mL of GNP, (b) 6 µL of SF (1mg/mL) in 0.66 µM FITC in 3 mL of GNP, (c) 10 µL of SF (1mg/mL) in 0.66 µM FITC in 3 mL of GNP, and (d) 400 µL of SF (1mg/mL) in 0.66 µM FITC in 3 mL of GNP.


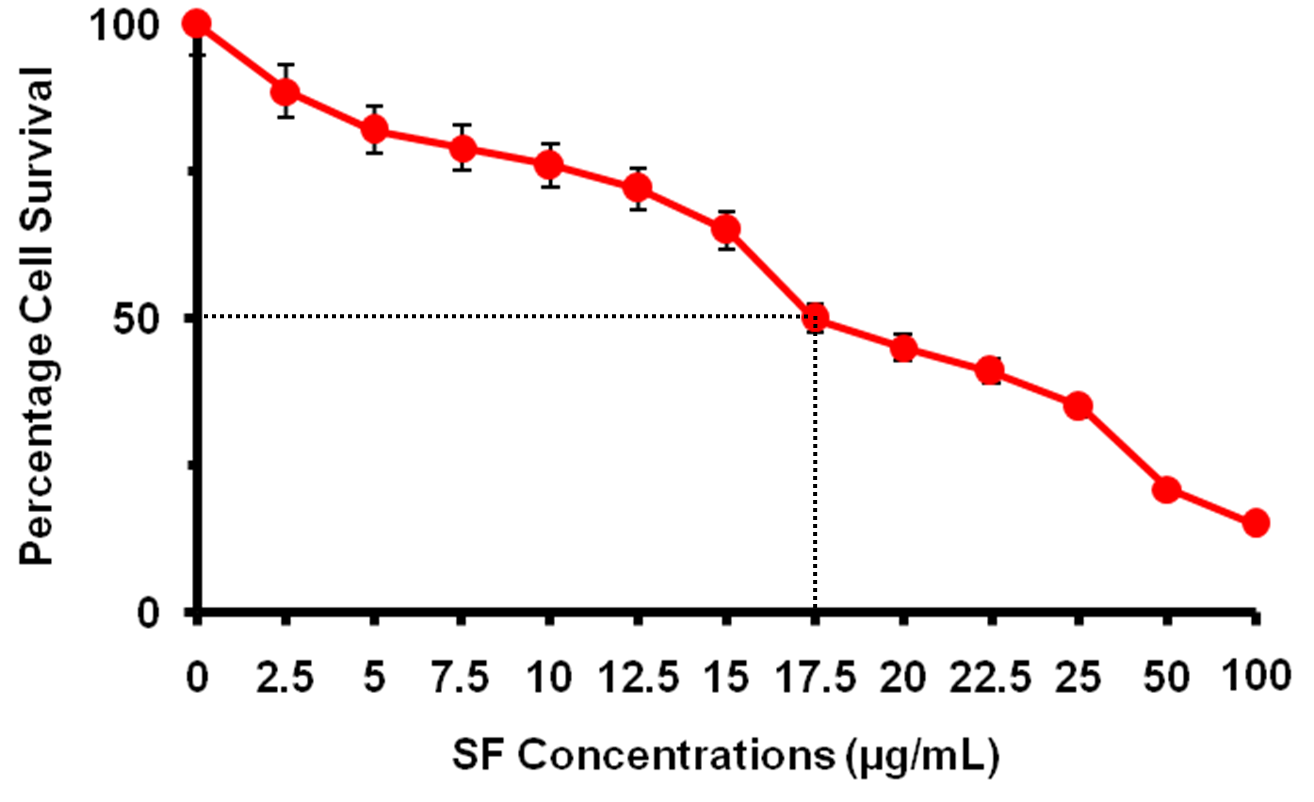


**Fig. S3.1** Determination of half-maximal (50%) inhibitory concentration (IC50) of SF-mediated cytotoxicity in HepG2 cells evaluated by treating HepG2 cells with increasing concentrations of SF (0-100µg/mL). Data are expressed as the mean±SEM of six replicates. Data shown are representative of three independent experiments.


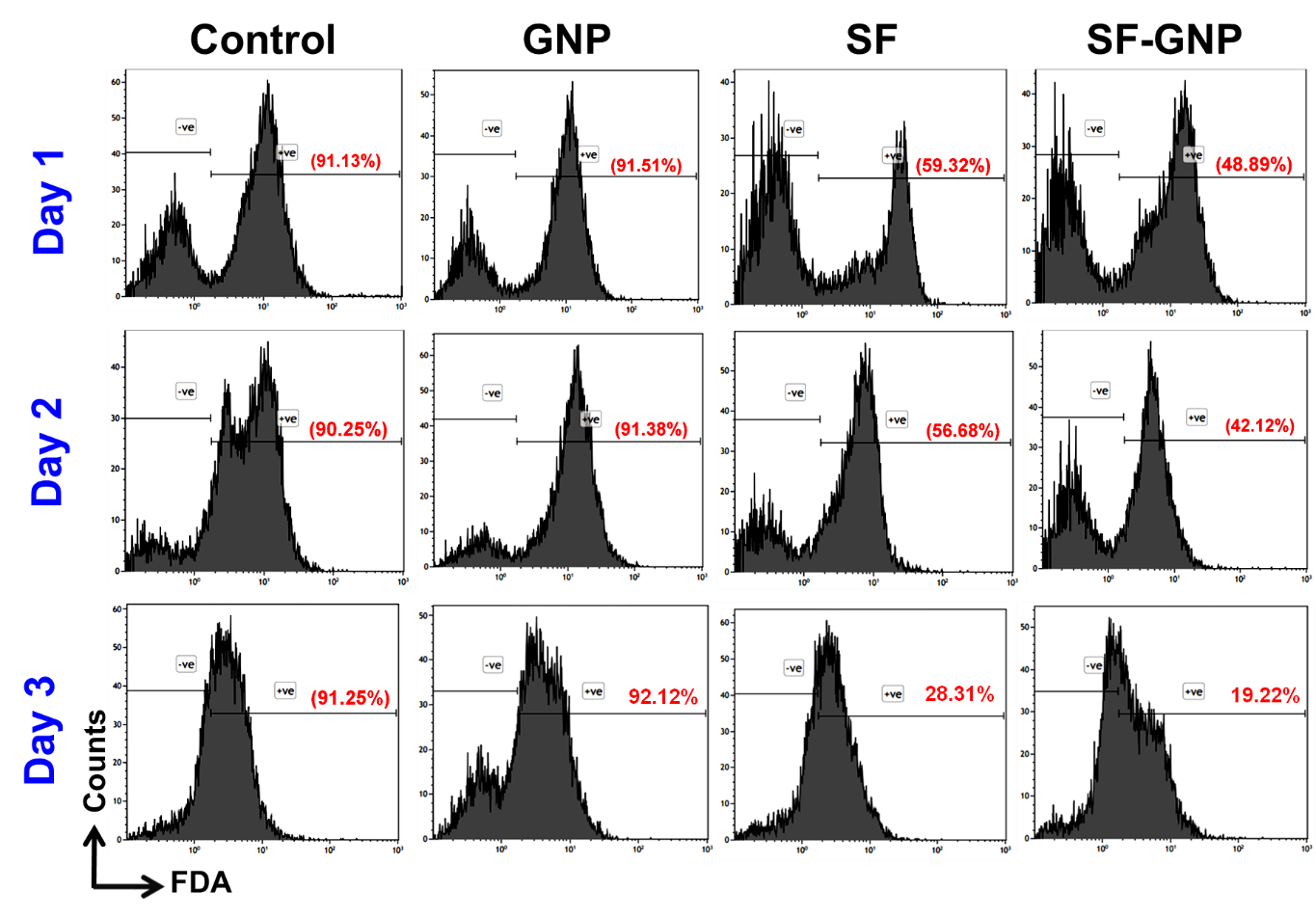


**Fig. S3.2** FDA flow cytometry analysis of HepG2 cells obtained from developing colonies at day 1, 2 and 3 post treatments with GNP did not show difference with controls whereas free SF treatment revealed approximately 40% decrease in cell survival which was further reduced more 20-30% after treatment with SF-GNP conjugate. Time dependent variation in percentage cell death was observed with free SF and SF-GNP conjugate treatment.


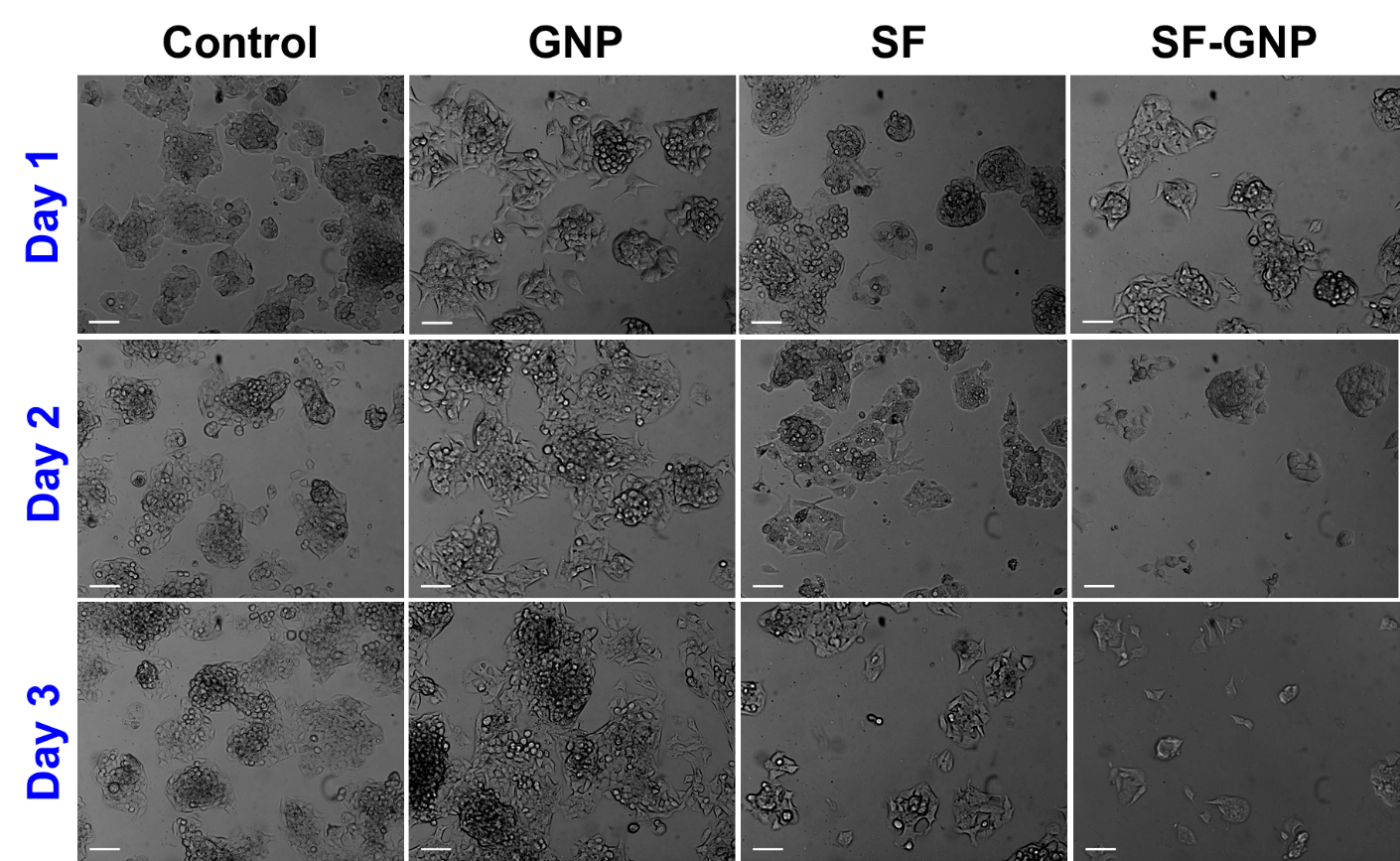


**Fig. S3.3** Optical microscopic observation for the effect of GNP, free SF and SF-GNP conjugate on growing HepG2 cell colonies number and size at day 1, 2 and 3 revealed time dependent significant decrease in SF-GNP treated group as compared to SF. GNP had no effect on the reduction of colony number or size with the time.


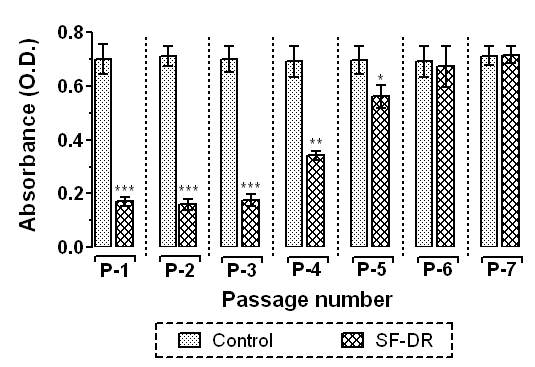


**Fig. S4.1** MTT absorbance value for HepG2 cells survival from passage 1 to 7 at day 3 during exposure to IC(50) of SF. The optical density at passage 6 and 7 was similar to the control groups. Therefore cells at passage 6 treated with SF were considered at SF resistant cells and used for further experimental purposes. Data are expressed as the mean±SEM of four replicates which are representative of five independent experiments.


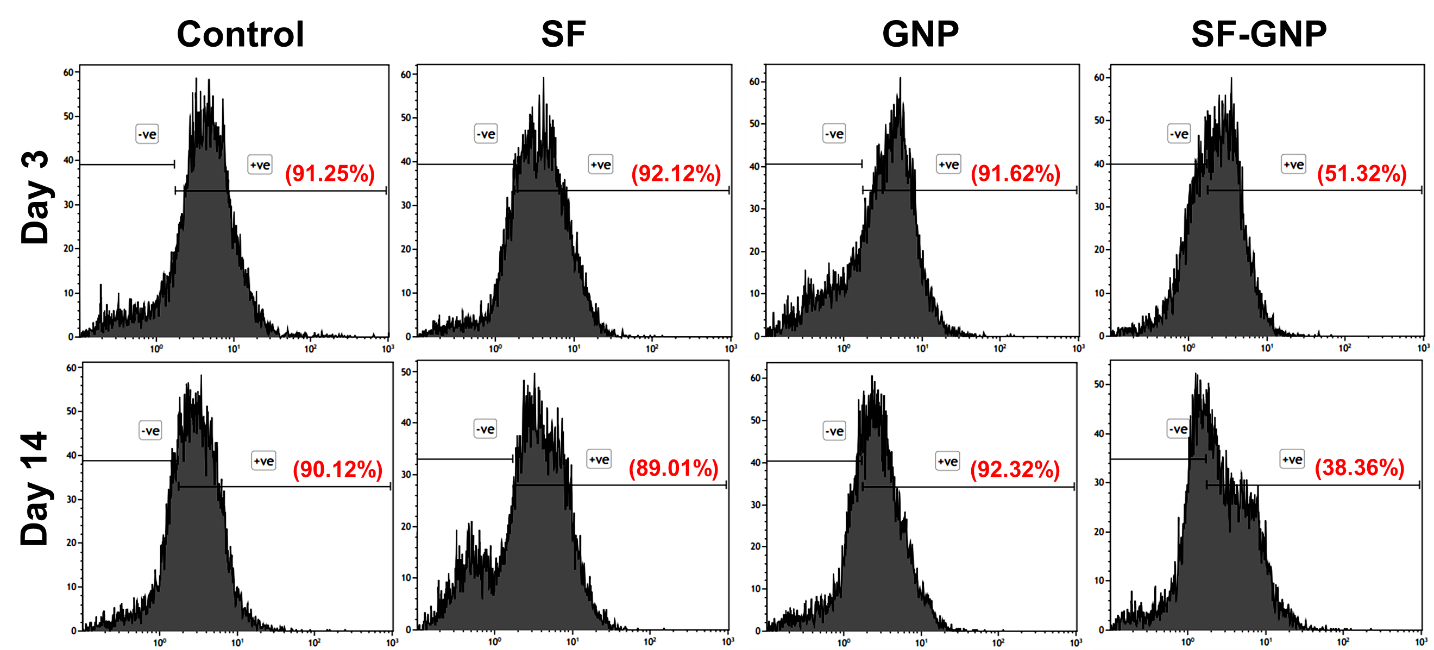


**Fig. S4.2** FDA flow cytometry analysis of SF resistant HepG2 cells obtained from developing colonies at day 3 and 14 post treatments with SF, GNP and SF-GNP conjugate. Approximately 50% at day 3 (p<0.01) and >60% cell death at day 14 (p<0.001) was observed after treatment with SF-GNP conjugate to SF resistant HepG2 cell colonies.


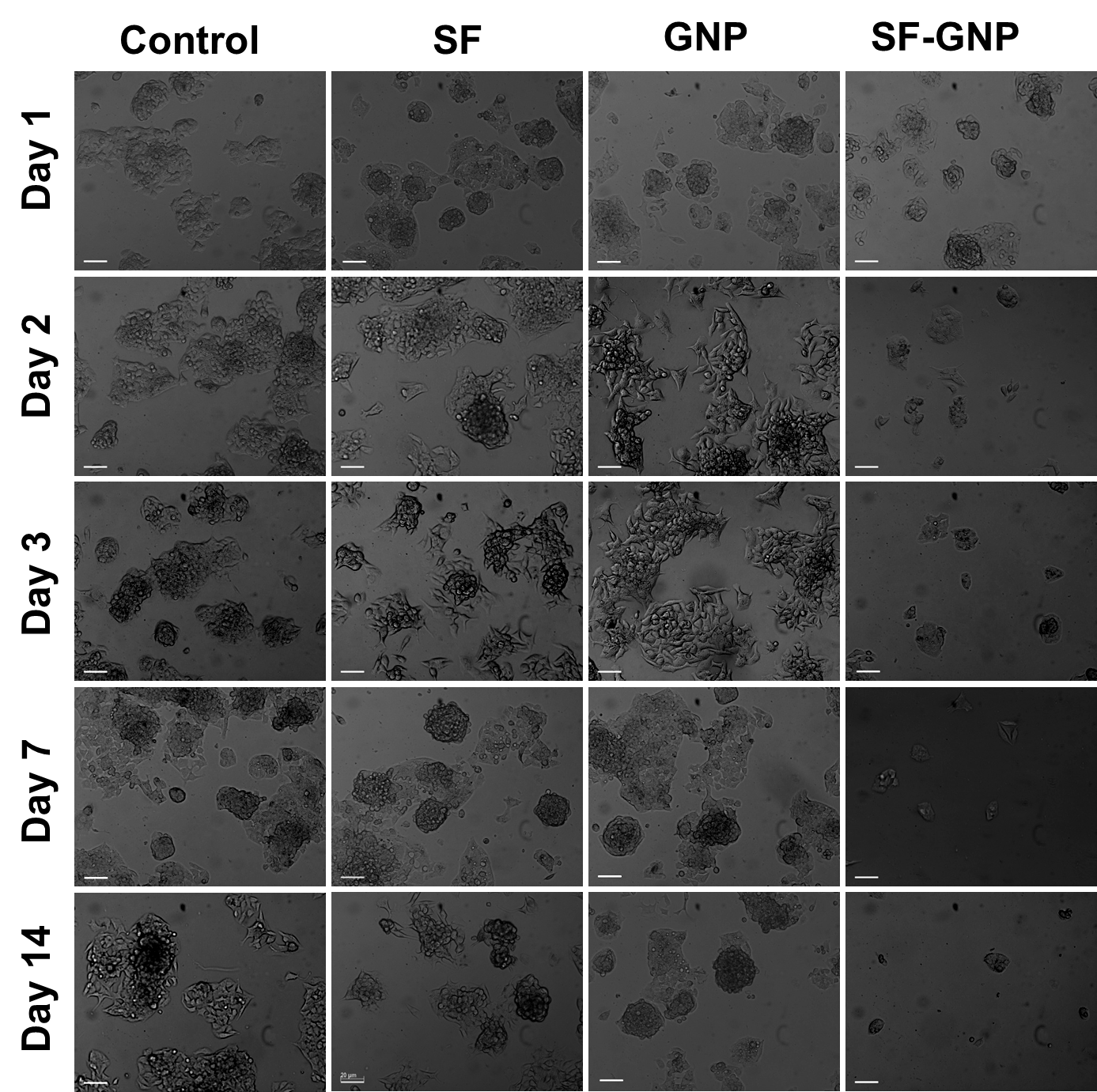


**Fig. S4.3** Optical microscopic observation of SF resistant HepG2 cell colonies number and size at day 1, 2, 3, 7 and 14 after treatments with SF-GNP conjugate showing the time dependent significant decrease as compared to other groups.


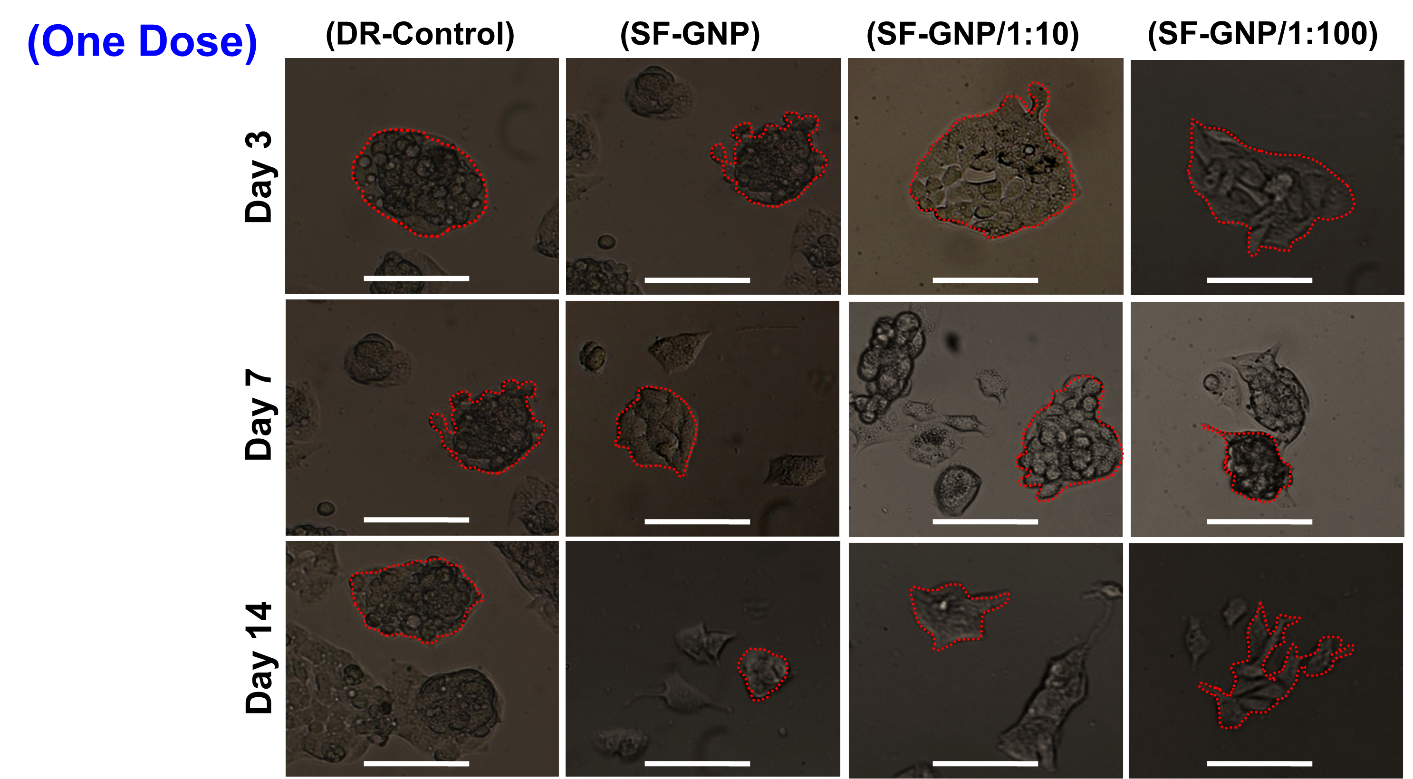


**Fig. S5.2** Optical microscopic observation of single dose effect of SF-GNP conjugate on growing SF resistant HepG2 cell colonies at day 3, 7 and 14


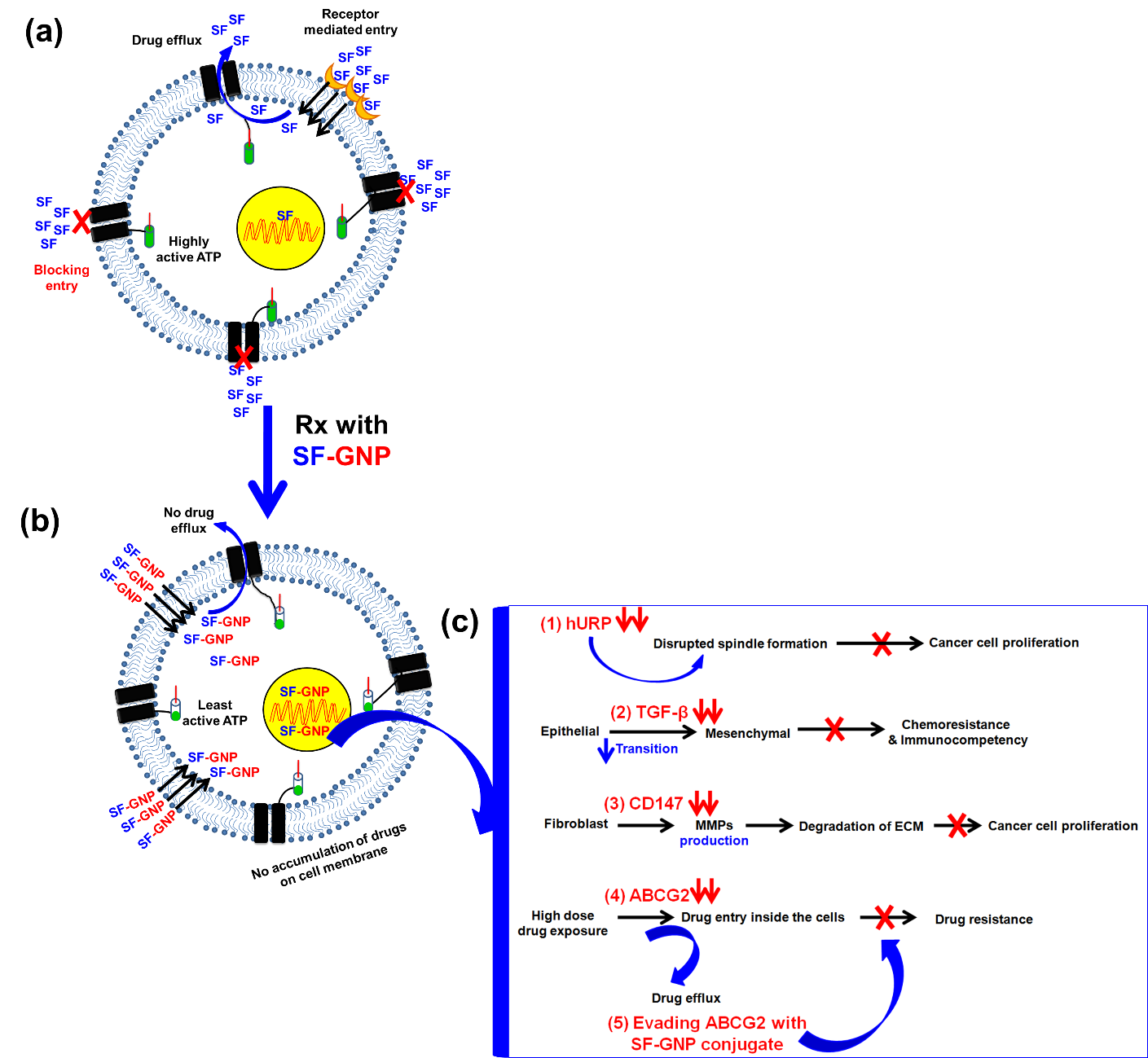


**Fig. S5.3** Mechanism by which SF-GNP conjugate contributes to additionally enhanced chemotherapeutic response in SF resistant HepG2 cells. (a) Common mechanism of SF resistance in cancer cells showing activation of membrane transport molecules which block the entry of SF molecules and efflux the overdose or continuous exposure of high dose of SF from the cells. (b, c) Proposed mechanism for SF-GNP entry into SF resistant cancer cells by evading membrane transporters resulting in reduced expression of ABCG2 which is not sufficient to block the entry of SF-GNP into the cells. In addition, the low dose of SF-GNP may also have one reason not to affect the ABCG2 which does not produce early SF resistance in cancer cells. Further, the down regulation of hURP may result in disrupted spindle formation during cancer cell division which could inhibit the proliferation of SF resistant HepG2 cells after treatment with SF-GNP. The declined expression of TGF-β transcript expression after SF-GNP treatment may decrease the EMT ultimately resulting in declined chemoresistance and immunocompetency of SF resistant HepG2 cells. Further, the decrease in the expression of CD147 results in enhanced MMPs production which may degrade the extracellular matrix (ECM) of cancer cells and decrease the proliferation of SF resistance HepG2 cells after treatment with SF-GNP conjugate.

**
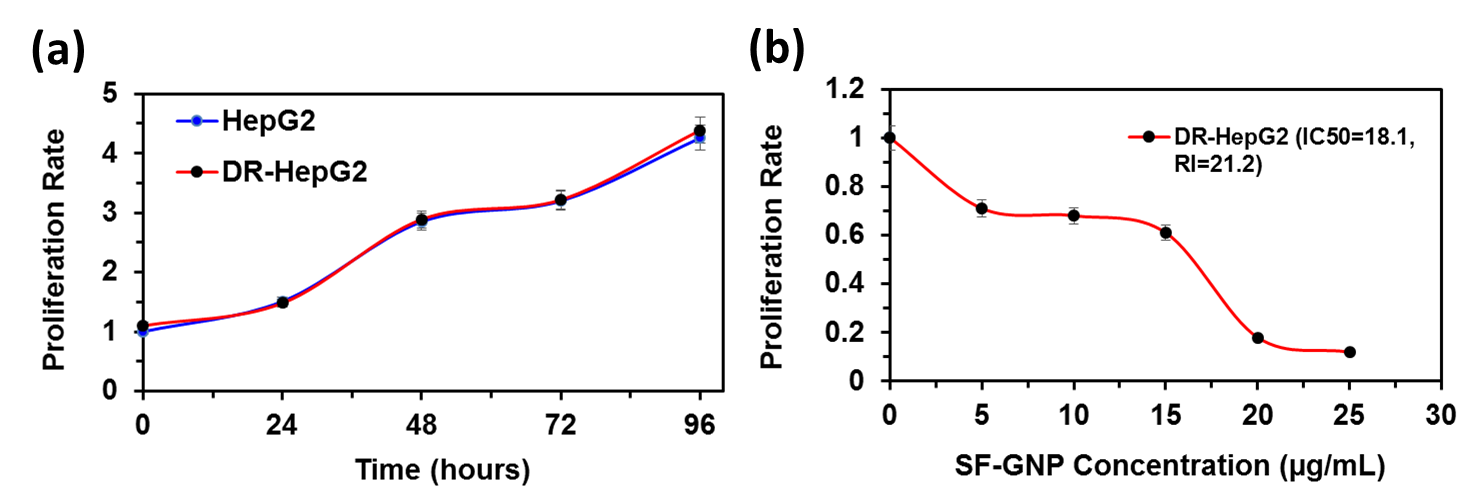
**

**Fig. S6.1** Resistance index in SF-resistant HepG2 cells (referred as DR-HepG2). (a) We did not observe any change in proliferation of both types of cells i. e. normal HepG2 and SF resistant HepG2. (b) However, the IC50 value of SF-GNP for SF-resistant HepG2 cells was found to be 18.1µg/mL with resistance index of 21.2 after 72h.

**
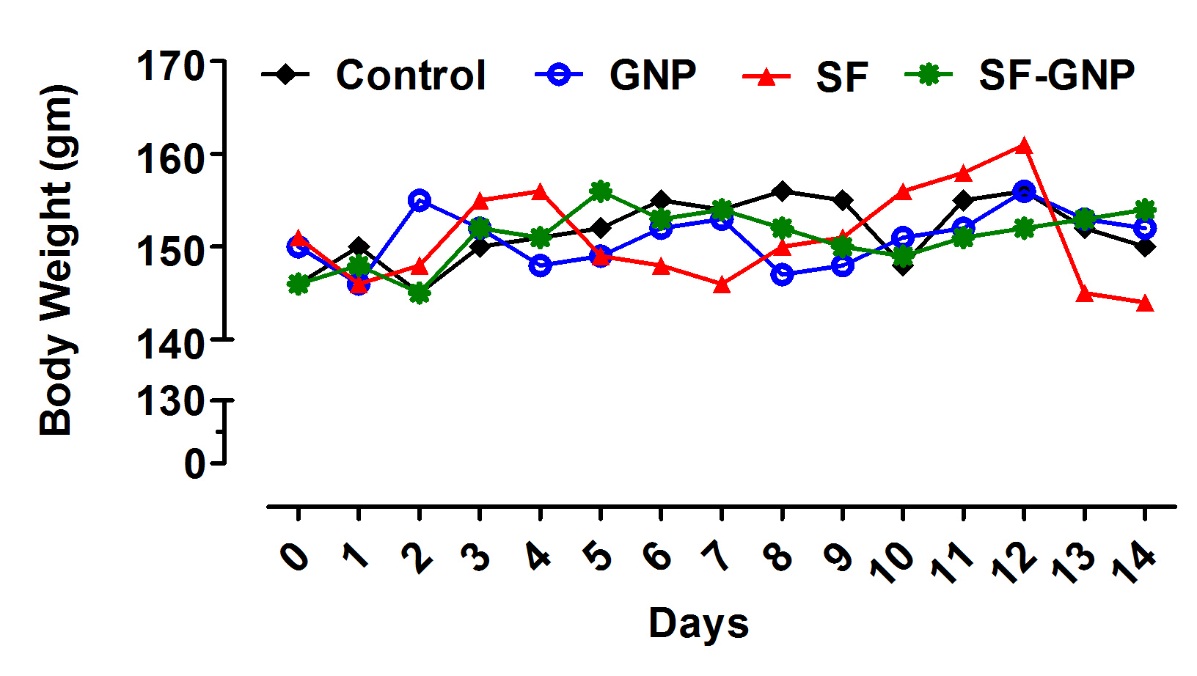
**

**Fig. S6.2** Body weight profile of animals in different groups did not show significant change for 14 days after intraperitoenal administration of GNP, free SF or SF-GNP


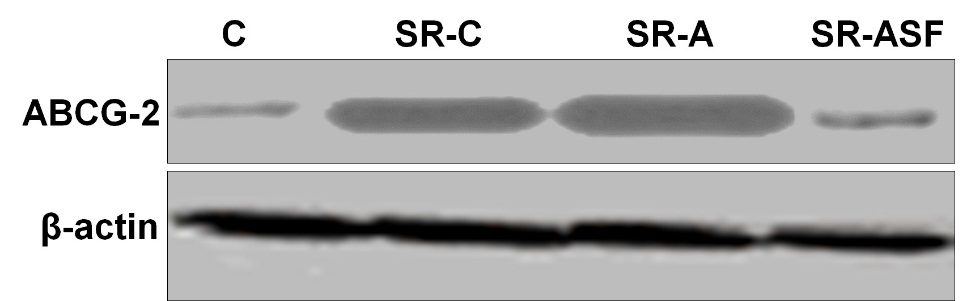


**Fig. S6.3** Western blot showing significant decrease in ABCG-2 levels after SF-GNP treatment to SF-resistant solid tumor colonies of HepG2 cells in soft agar at day 14


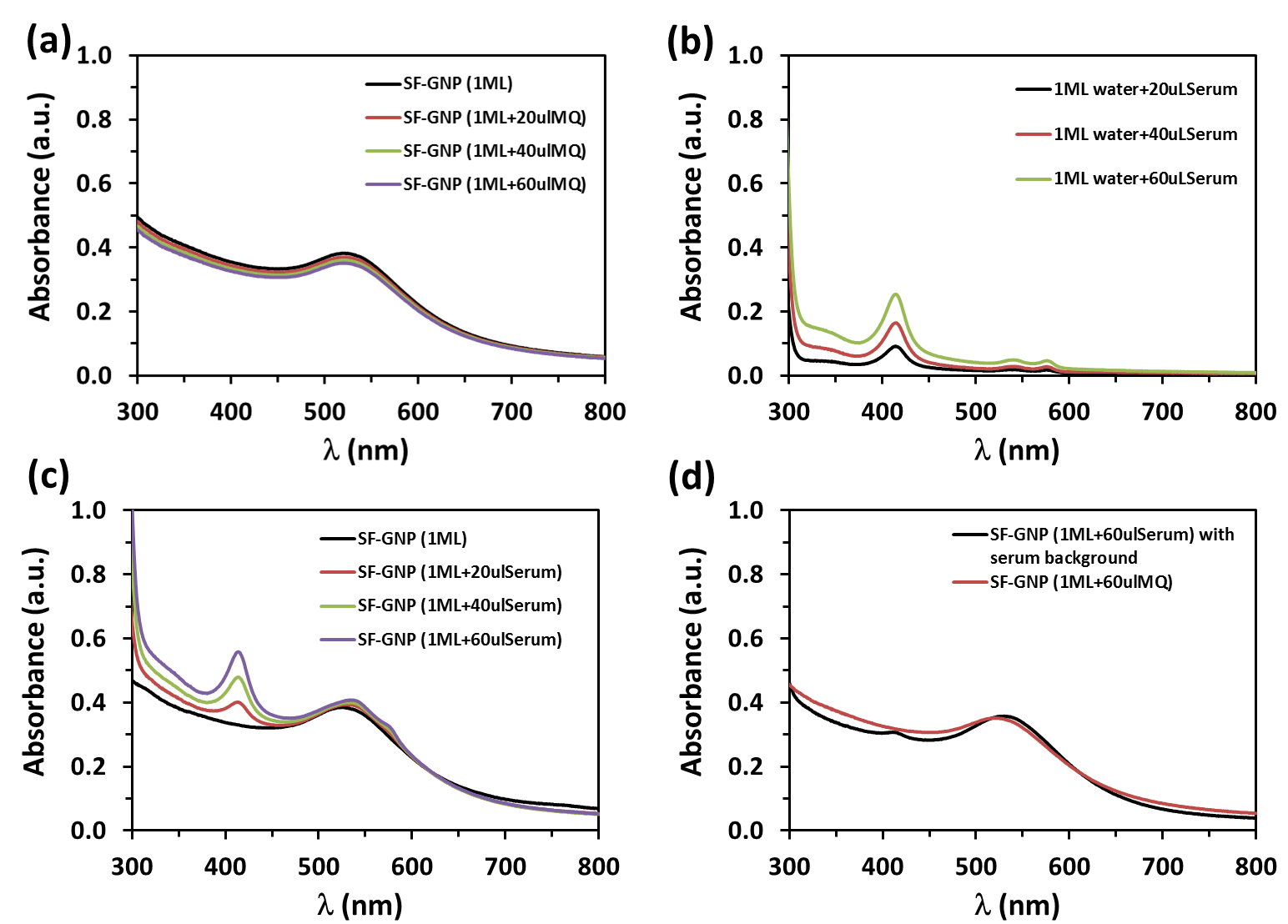


**Fig. S6.4** Serum stability analysis of SF-GNP nanoconjugate did not any significant change under the effect of variable concentrations of serum addition

**Table: S1.1** Linearity response of SF in HPLC

| **S. No** | **Concentration**  **in µg/mL** | **Area of SF** | **S. No** | **Concentration**  **in µg/mL** | **Area of SF** |
| --- | --- | --- | --- | --- | --- |
| 1 | 1 | 62220 | 15 | 60 | 3882438 |
| 2 | 2 | 122174 | 16 | 70 | 4233976 |
| 3 | 3 | 176026 | 17 | 80 | 4849649 |
| 4 | 4 | 249283 | 18 | 90 | 5433467 |
| 5 | 5 | 304727 | 19 | 100 | 6452150 |
| 6 | 6 | 370575 | 20 | 200 | 8503597 |
| 7 | 7 | 447598 | 21 | 300 | 12683152 |
| 8 | 8 | 487939 | 22 | 400 | 16713501 |
| 9 | 9 | 544290 | 23 | 500 | 21884767 |
| 10 | 10 | 598027 | 24 | 700 | 29136801 |
| 11 | 20 | 1209456 | 25 | 800 | 33417592 |
| 12 | 30 | 1831602 | 26 | 900 | 37309516 |
| 13 | 40 | 2441506 | 27 | 1000 | 40991857 |
| 14 | 50 | 3067913 |  |  |  |

**Table: S2.1** Mean±SD of values for hematological parameters did not show the significant variation in male Wister rats at day 3 post IP administrations of GNP, free SF and SF-GNP conjugate

**Table: S2.2** Mean±SD of values for hematological parameters did not show the significant variation in male Wister rats at day 7 post IP administrations of GNP, free SF and SF-GNP conjugate

**Table: S2.3** Mean±SD of values for hematological parameters did not show the significant variation in male Wister rats at day 14 post IP administrations of GNP, free SF and SF-GNP conjugate

**Table S3.1** Mean±SD of values for biochemical analysis performed to identify the functional response of vital organs, serum LFT parameters albumin ALP, bilirubin, SGOT and SGPT and RFT parameters (Urea and Creatinine) showed normal range at day 3, 7 and 14 post-IP injection of GNP, free SF and SF-GNP conjugate in Wister rats.

**Liver function test (RFT)**

**Albumin**

| **Time** | **Control** | **GNP** | **SF** | **SF-GNP** |
| --- | --- | --- | --- | --- |
| **Day 3** | 3.072± 0.2485 | 3.064±0.1567 | 2.920±0.1372 | 3.284±0.1505 |
| **Day 7** | 3.354±0.2558 | 3.001±0.2226 | 3.223±0.2545 | 2.910±0.1857 |
| **Day 14** | 2.850±0.2240 | 3.036±0.2651 | 2.530±0.1373 | 3.519±0.3886 |

**ALP**

| **Time** | **Control** | **GNP** | **SF** | **SF-GNP** |
| --- | --- | --- | --- | --- |
| **Day 3** | 96.75±11.69 | 104.6±7.374 | 102.4±14.11 | 98.67±13.17 |
| **Day 7** | 97.81±9.763 | 84.77±9.853 | 89.58±8.851 | 94.67±13.36 |
| **Day 14** | 105.5±7.127 | 96.85±10.01 | 99.42±14.24 | 113.0±8.175 |

**Bilirubin**

| **Time** | **Control** | **GNP** | **SF** | **SF-GNP** |
| --- | --- | --- | --- | --- |
| **Day 3** | 0.2727±0.03765 | 0.2671±0.02923 | 0.2587±0.03335 | 0.2530±0.02636 |
| **Day 7** | 0.2672±0.02682 | 0.2696±0.02717 | 0.2657±0.02613 | 0.2449±0.03087 |
| **Day 14** | 0.2983±0.02468 | 0.2889±0.03933 | 0.2557±0.03644 | 0.2835±0.02795 |

**SGOT**

| **Time** | **Control** | **GNP** | **SF** | **SF-GNP** |
| --- | --- | --- | --- | --- |
| **Day 3** | 49.47±3.931 | 66.77±7.395 | 52.14±2.736 | 50.60±5.187 |
| **Day 7** | 54.48±7.405 | 60.05±7.862 | 56.73±6.281 | 54.95±7.068 |
| **Day 14** | 58.02±6.147 | 65.76±1.511 | 42.32±5.034 | 42.95±3.103 |

**SGPT**

| **Time** | **Control** | **GNP** | **SF** | **SF-GNP** |
| --- | --- | --- | --- | --- |
| **Day 3** | 23.57±2.726 | 25.57±2.076 | 25.33±2.351 | 23.68±2.450 |
| **Day 7** | 20.83±1.408 | 21.70±1.407 | 20.13±2.982 | 19.99±2.814 |
| **Day 14** | 21.37±2.697 | 20.81±2.284 | 27.35±1.906 | 22.77±4.367 |

**Renal function test (RFT)**

**Urea**

| **Time** | **Control** | **GNP** | **SF** | **SF-GNP** |
| --- | --- | --- | --- | --- |
| **Day 3** | 18.25±2.253 | 16.99±1.578 | 17.54±2.203 | 16.30±1.387 |
| **Day 7** | 18.69±1.034 | 18.14±1.607 | 21.85±1.103 | 19.35±1.261 |
| **Day 14** | 16.14±0.8221 | 19.52±0.9826 | 19.82±3.131 | 16.14±2.006 |

**Creatinine**

| **Time** | **Control** | **GNP** | **SF** | **SF-GNP** |
| --- | --- | --- | --- | --- |
| **Day 3** | 0.5546±0.02481 | 0.5480±0.03800 | 0.5389±0.03299 | 0.3983±0.02356 |
| **Day 7** | 0.6703±0.06122 | 0.6687±0.04176 | 0.7109±0.03664 | 0.5541±0.08814 |
| **Day 14** | 0.6579±0.05464 | 0.6331±0.02560 | 0.5381±0.08148 | 0.5829±0.07161 |
